# A Carbon Capture and Utilization Process for the Production of Solid Carbon Materials from Atmospheric CO_2_ – Part 1: Process Performance

**DOI:** 10.1002/cssc.202401779

**Published:** 2024-11-12

**Authors:** Neele Uhlenbruck, Peter Pfeifer, Benjamin Dietrich, Christoph M. Hofberger, Ralf Krumholz, Antonio Saxler, Linus Schulz, Leonid Stoppel, Thomas Wetzel

**Affiliations:** ^1^ Institute for Thermal Energy Technology and Safety Karlsruhe Institute of Technology Hermann-von-Helmholtz-Platz 1 76344 Eggenstein-Leopoldshafen Germany; ^2^ INERATEC GmbH Siemensallee 84 76187 Karlsruhe Germany; ^3^ Institute of Thermal Process Engineering Karlsruhe Institute of Technology Kaiserstr. 12 76131 Karlsruhe Germany

**Keywords:** Carbon storage, Hydrocarbons, Methanation, Methane pyrolysis, Liquid metal

## Abstract

The successful operation of a process that converts atmospheric CO_2_ into solid carbon products is presented as an alternative to fossil based solid carbon production. In a first step, CO_2_ is removed from the atmosphere by a direct air capture (DAC) unit. The gas is then mixed with hydrogen and enters a methanation unit. Depending on the operation conditions, gas mixtures consisting of mainly methane with either H_2_ or CO_2_ as side‐component are obtained. After precipitating the water formed during the methanation step, the remaining gas mixture is fed into a bubble column reactor filled with liquid tin. During the rise of the gas bubbles, methane is thermally split up into hydrogen and solid carbon. The latter is continuously removed from the liquid metal surface as a fine powder by pneumatic conveying. This article is the first of two articles, focusing on the performance of the methanation and methane pyrolysis steps. The experimental results are complemented by thermodynamic analyses and reaction modelling. A detailed analysis of the solid carbon product of the process is presented in the second part.

## Graphical abstract

Atmospheric CO_2_ was successfully converted into solid carbon products via a novel process, which combines direct air capture, catalytic methanation and liquid metal‐based methane pyrolysis. The influence of impurities and operation parameters on the process performance regarding conversions and product yields is analyzed for the overall process and for the individual process steps methanation and pyrolysis.

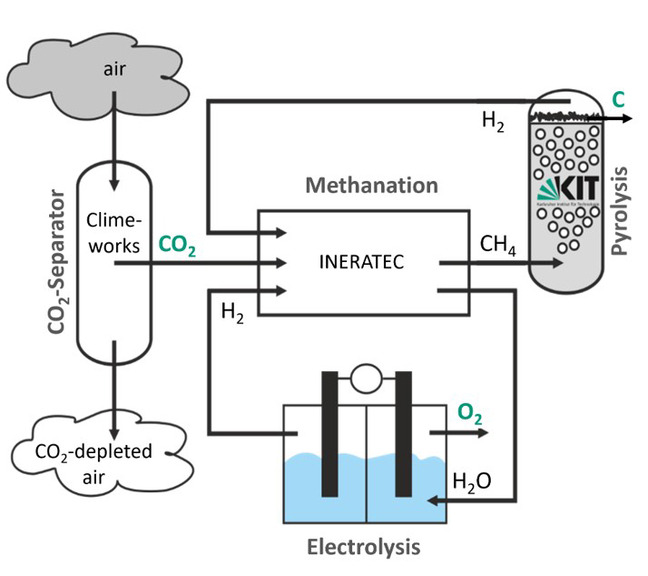

## 1 Introduction and Theoretical Background

A circular economy based on renewable energy that uses CO_2_ as an alternative to conventional fossil sources to synthesize fuels and chemicals has been the subject of many studies.[^1^, ^2^, ^3^] However, they do not address the conversion of CO_2_ into solid carbonaceous products although many commercial carbon products, e. g., carbon blacks and carbon fibers, are currently fossil based.[^4^, ^5^, ^6^] Research activities aiming at the conversion of fossil precursors into carbon materials are also still ongoing.[^7^, ^8^] During the past years, some processes have been presented that instead convert CO_2_ into various solid carbon materials. Depending on the process conditions, molten carbonate electrolysis results in the synthesis of Carbon Nano‐Onions (CNO),^[9]^ Carbon Nanotubes (CNT)[^10^, ^11^, ^12^] and further carbon morphologies.[^10^, ^11^, ^12^, ^13^, ^14^] In a different approach, Liang et al.^[15]^ synthesized graphite flakes via the reaction of CO_2_ and LiAlH_4_. However, research regarding Carbon Capture and Utilization (CCU) processes that aim at the production of solid carbon is still scarce compared to that focusing on the production of synthetic fuels or chemicals. In this study, we present the successful realization of a new process for the conversion of CO_2_ into solid carbon materials. The process route developed here pursues the idea of synthesizing methane from atmospheric CO_2_ using hydrogen in a closed loop. In the final process step, the so‐called liquid metal‐based methane pyrolysis, CH_4_ is then broken down into its components, hydrogen, and carbon. This article is the first of two parts, reporting the results concerning the performance of the methanation and the pyrolysis step under various process conditions. A special focus lies on the effects caused by impurities in the product gases of the DAC and methanation unit. Regarding methanation, catalyst performance with toluene and oxygen as impurities is analyzed. With respect to methane pyrolysis, unconverted H_2_ and CO_2_ from the preceding methanation step are the most relevant impurities and their effect on pyrolysis performance is investigated at various temperature levels. Besides, the influence of methane dilution on pyrolysis is analyzed as no pure methane is produced during the methanation step. Methane conversions and hydrogen yields obtained in bubble column reactors filled with liquid metal (LM) have already been addressed in several studies.[^16^, ^17^, ^18^, ^19^, ^20^, ^21^, ^22^, ^23^, ^24^, ^25^] However, carbon yields have only been addressed by a few authors. Tang et al.^[26]^ obtained carbon yields of close to 60 % when they investigated the pyrolysis of 0.5 to 5 vol.% methane in molten copper for temperatures ranging from 1150 °C to 1450 °C. Using a reactor filled with liquid tin, Qiao et al.^[27]^ achieved a carbon yield of 70 % with 4 vol.% CH_4_ at 1250 °C. Both studies focus on the pyrolysis of strongly diluted methane at high temperatures to synthesize graphene or graphite nanoplatelets. In this article, we analyze the carbon yields that can be obtained for methane concentrations of up to 87 vol.% at pyrolysis temperatures ranging from 1000 °C to 1100 °C. To the best of our knowledge, carbon yields have not yet been reported for comparable synthesis conditions in molten media reactors. In addition to reporting carbon yields, the present study demonstrates the successful continuous removal of carbon from the pyrolysis reactor via pneumatic conveying. Several options for the continuous removal of the carbon product from reactors filled with molten media have been suggested by other authors[^16^, ^19^, ^20^, ^28^] but have not been implemented. An analysis of how the addition of an inert carrier gas above the LM surface quenches the pyrolysis reactions and affects the pyrolysis process is included. The successful quenching of the pyrolysis gas above the LM surface and the continuous removal of the carbon powder lay the foundation for a thorough analysis of the carbon products synthesized under various pyrolysis conditions. A detailed characterization of these solid carbon products is given in the second part.^[29]^

### 1.1 NECOC Process

**Figure 1:**
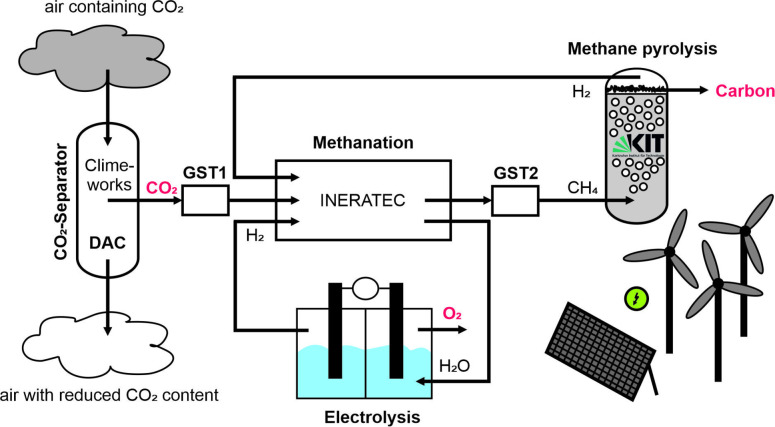
Scheme of the coupled CCU process that converts atmospheric CO_2_ into solid carbon via direct air capture (DAC), methanation and methane pyrolysis. After the individual process steps, the product gas is passed into gas storage tanks (GST). Recycling of hydrogen from methane pyrolysis and an additional electrolyzer create a closed hydrogen loop in an ideal process.

The process shown in Figure 1, which has been demonstrated successfully at the Karlsruhe Institute of Technology (KIT), combines several steps to convert atmospheric CO_2_ into solid carbon powder. It has the potential to reduce the atmospheric CO_2_ concentration, originating partly from anthropogenic emissions, in two ways: by actively consuming CO_2_ as a feedstock and by providing a cleaner alternative to current fossil‐based carbon synthesis routes. In a first step, developed and operated by Climeworks Deutschland GmbH (referred to as Climeworks in the following), CO_2_ is removed from the air via direct air capture (DAC). A catalytic methanation step, developed and operated by INERATEC GmbH (referred to as INERATEC in the following), then converts CO_2_ and hydrogen into methane and water. After separating the water in a cold trap, CH_4_ is thermally split up into hydrogen and a solid carbon powder in a liquid metal filled bubble column reactor, which was developed at KIT.[^16^, ^17^] The H_2_ produced during the CH_4_ pyrolysis step is recycled to the methanation unit. To achieve a closed hydrogen loop, i. e., minimizing external supply of hydrogen, the water product of the methanation step is split in an electrolyzer and fed back to the methanation. The electrolyzer and the hydrogen recycling were not part of the present study, though, which focused on the experimental coupling of the sequence DAC‐methanation‐CH_4_‐pyrolysis.

### 1.2 Direct Air Capture

Concentrated CO_2_ is obtained from the atmosphere via a three‐step DAC process developed by Climeworks.^[30]^ First, ambient air passes through a collector unit. Once the collector is saturated with CO_2_, the air stream is cut off and a temperature increase to about 100 °C results in the release of the captured gas.^[30]^ The gas is then, after condensation of water at 5 °C passed on via a vacuum pump into a gas balloon at ambient conditions. Oxygen concentration was monitored to be less than 800 ppm as the CO_2_ was then compressed and sent into a first gas storage tank (GST1 in Figure 1) at 10 bar for provision into the methanation unit. Nickel catalysts for methanation are typically sensitive to oxygen. Other gas components or organic residues from the adsorption process were not monitored at this process step and therefore it was decided to investigate such components in the methanation unit as individual co‐feed (see subsection 2.1).

### 1.3 Methanation of CO_2_

The Sabatier reaction, given by Equation (1) and performed in the INERATEC module is a strongly exothermic equilibrium reaction and requires a good control over the released heat to obtain high conversions with an elongated catalyst lifetime. Otherwise, a clean methane stream can only be obtained via adsorption or cryo‐processes which are not intended here since the overall dissociation energy for CO_2_ in the process should be kept minimal.

(1) $$CO_{2}\left(g\right)+4{\;H}_{2}\left(g\right)↔\;{2\;H}_{2}O\left(g\right)+{CH}_{4}\left(g\right)\;\Delta H_{R}^{0}=-165\;\;kJ/mol\;{CO}_{2}$$

During the Sabatier reaction also the reversed water gas shift (rWGS) reaction can occur as an intermediate reaction, resulting in carbon monoxide formation according to Equation (2). This together with a possible CO_2_ and/or H_2_ conversion of less than 100 % could be crucial for the subsequent methane pyrolysis as neither the addition of CO, CO_2_ or H_2_ to the pyrolysis feedstock has been studied before in a tin filled bubble column reactor.

(2) $$CO_{2}\left(g\right)+{\;H}_{2}\left(g\right)↔\;CO\left(g\right)+H_{2}O\left(g\right)\;\Delta H_{R}^{0}=41\;\;kJ/mol\;{CO}_{2}$$

### 1.4 Methane Pyrolysis

The conversion of methane to hydrogen and solid carbon takes place via a long and complex reaction mechanism.[^31^, ^32^] The initial and rate determining step is assumed to be the formation of methyl radicals, which is then followed by a stepwise dehydrogenation via ethane, ethylene and acetylene.^[33]^ Then the formation of larger molecules leads to a further reduction of the molecular H/C ratio. The intermediates may form either solid carbon on surfaces via a chemical vapor deposition mechanism^[31]^ or further increase in size while releasing more hydrogen until soot particles are formed and grow.[^34^, ^35^] Equation (3) gives the simplified net equation of methane pyrolysis without intermediate steps, byproduct formation or a distinction between different types of solid carbon.

(3) $$CH_{4}\left(g\right)↔2\;H_{2}\;\left(g\right)+C\;\left(s\right)\Delta H_{R}^{0}=74.8\;\;kJ/mol\;CH_{4}$$

When CO_2_, CO and/or humidity are present in the methanation product gas, further side reactions are expected to occur in the pyrolysis reactor. At the high methane pyrolysis temperatures of more than 1000 °C, the gasification of carbon by both CO_2_ and H_2_O must be taken into consideration, as given in Equation (4) and Equation (5). CO_2_ typically starts to react with carbon at significant rates in a temperature range between 800 and 900 °C.[^36^, ^37^, ^38^] Reaction kinetics vary depending on the carbon properties and impurities,[^39^, ^40^] though, and tend to be faster for steam gasification than for CO_2_.[^39^, ^40^, ^41^, ^42^]

(4) $$C\;\left(s\right)\;+\;CO_{2}\;\left(g\right)\;↔2\;CO\;\left(g\right)\Delta H_{R}^{0}=172.5\;\;kJ/mol\;CO_{2}$$

(5) $$C\;\left(s\right)\;+\;H_{2}O\;\left(g\right)↔CO\;\left(g\right)+H_{2}\;\left(g\right)\Delta H_{R}^{0}=131.3\;\;kJ/mol\;H_{2}O$$

Also, the rWGS reaction (Equation (2)) can occur under the reaction conditions of the pyrolysis step.^[43]^ Besides, dry reforming of methane (DRM) and steam reforming of methane (SRM), given by Equation (6) and Equation (7) respectively, are further possible reactions when CO_2_ or water are present in the pyrolysis feed gas.

(6) $$CH_{4}\left(g\right)\;+{CO}_{2}\left(g\right)↔\;{2\;H}_{2}\;\left(g\right)+2\;CO\;\left(g\right)\Delta H_{R}^{0}=247.3\;\;kJ/mol\;CH_{4}$$

(7) $$CH_{4}\left(g\right)\;+H_{2}O\left(g\right)↔\;{3\;H}_{2}\;\left(g\right)+CO\;\left(g\right)\Delta H_{R}^{0}=206.1\;\;kJ/mol\;CH_{4}$$

### 1.5 Coupled Operation

The single process steps DAC, methanation and methane pyrolysis by themselves have been tested before, as reported elsewhere for methanation[^44^, ^45^] and for methane pyrolysis.[^16^, ^46^] However, results of the coupled operation of these processes are presented in this article for the first time. During the implementation of the coupled process, the following challenges had to be overcome:

- Using atmospheric CO_2_ from DAC, which includes some impurities.
- Producing an almost CO‐free (≤ 0.01 vol.%) methane with negligible CO_2_ (<0.2 vol. %) for further pyrolysis.
- Demonstrating stable pyrolysis operation with feed gas containing up to 5.6 % CO_2_ and up to 0.1 % CO (methanation conditions differing from b).
- Thermally splitting methane gas mixtures from CO_2_ methanation that include not only unconverted CO_2_ and/or H_2_ but also impurities from previous DAC and residual humidity from the methanation stages (condensation at 5 °C and 8 bar(a) results in minimum ~0.1 vol.% of product water left in the gas stream based on equilibrium vapor pressure).

Besides, the two‐stage methanation unit has been adjusted in comparison to an earlier study^[45]^ by applying a much smaller flow rate due to the combination with the pyrolysis unit.

A significant improvement of the pyrolysis system was achieved by modifying the reactor so that the synthesized carbon powder could be removed continuously via pneumatic conveying during pyrolysis operation. Thus, carbon yields for various process conditions were obtained. To the best of our knowledge, the effect of temperatures in the range of 1000 °C to 1100 °C and methane concentrations between 25 vol.% and 87 vol.% on the carbon yield has not been studied before for liquid metal filled pyrolysis reactors. The continuous removal of carbon from the tin surface also allowed new insights into the role of the upper part of the pyrolysis reactor, which has not been studied in detail before. The experimental detection of acetylene in the pyrolysis gas is discussed and compared to model results. It is in contrast to previous results reported for comparable systems, where the product gas contained ethane instead.[^16^, ^18^] In addition to carbon yields, also methane conversions and hydrogen yields are evaluated for various pyrolysis temperatures and methane concentrations in the reactant gas. This analysis is further complemented by a reevaluation of previously obtained data^[46]^ for pure CH_4_ and CH_4_‐N_2_ mixtures. By comparing this data with the pyrolysis performance in the coupled process, possible effects of CO_2_ and/ or H_2_ in the methane mixture are analyzed.

## 2 Experimental

### 2.1 Catalytic Methanation of Carbon Dioxide

As a result of the relatively low flow rates of the present study, the cooling requirement was massively reduced compared to the methanation setup described by Guilera et al.^[45]^ Thus, the process can be described according to Figure S1 in the supplementary information (SI) as a two‐stage process where only the first reactor stage is cooled with some nitrogen instead of evaporation of water, which is the otherwise industrial concept for larger scale. The second stage, due to the low flow rates and the low residual conversion effort, does not require cooling at all and is solely heated to reaction conditions. Details about the reactor stages can be found elsewhere.^[45]^ For only investigating methanation, CO_2_ was either used from the DAC module or from gas bottles (grade 3.0) to perform studies on catalyst stability in the presence of impurities. As the electrolyzer shown in Figure 1 was not part of the actual experimental setup and hydrogen was not recycled from the pyrolysis unit, hydrogen needed for the methanation of CO_2_ was taken from bottles with identical gas grade of 3.0. In contrast to interconnected operation with pyrolysis, nitrogen was used as balance in the reaction and to determine conversion levels or residence time in the overall setup. The applied catalyst in the reactor stages was delivered by IREC and is based on γ‐Al_2_O_3_ impregnated with 26 wt.–% Nickel (Ni) and 21 wt.–% Cerium oxide (CeO_2_). Gas hourly space velocity (GHSV) was calculated from the standard volume flow divided by the reaction volume (volume filled with catalyst). To test the effects of oxygen and toluene as potential impurities in the CO_2_ feed, low levels of each substance have been added to the gas entering the first methanation stage in separate experiments. The applied levels of oxygen and toluene are given in Table S1 in the SI.

*Thermodynamic calculations* have been performed by minimization of the Gibbs’ free enthalpy in Matlab® using Shomate type equations for enthalpy and entropy. The corresponding parameters for the Shomate equations for the individual species have been obtained from the NIST Database.

*Analysis of Permanent Gases* (H_2_, CO_2_, CO, CH_4_) was done via an ND‐IR system EL3020 supplied by ABB.

*BET surface area calculation* was done from N_2_ physisorption on the catalyst according to DIN ISO 9277 : 2022.

### 2.2 Methane Pyrolysis

Figure S2 in the SI shows the setup of the methane pyrolysis facility. The pyrolysis reactor is a quartz tube (inner diameter *d*_i_=40 mm, length *L*=1300 mm) with a single orifice (*d*_O_=0.5 mm) at the bottom, through which the reactant gas mixture enters. A detailed description of the reactor setup is given elsewhere.[^16^, ^46^] The gas flows into the reactor are controlled by mass flow controllers (MFC) with relative accuracies of 0.5 %, which were calibrated for pure methane (reactant gas) and pure argon (carrier gas). The set value of the methane MFC was corrected to obtain the actual flow of the reactant gas ${\dot{V}}_{reac}$ based on information provided by the manufacturer for the gas mixtures employed in this study (see SI for details). The reactor is filled with liquid tin (99.99 %) to a height of approx. 1020 mm. The liquid tin serves as a heat transfer fluid. It also prevents reactor clogging as elaborated in previous studies.[^16^, ^46^] When a gas flow is applied to the reactor inlet, gas bubbles form at the orifice. While they rise in the liquid metal (LM) methane decomposes due to the high temperatures. An electric tube furnace (not depicted in Figure S2) heats the reactor from the outside to keep the LM at the center of the reactor tube at either approx. 1000 °C, 1050 °C or 1100 °C. Eleven type K thermocouples (TC) are positioned along the vertical axis of the reactor tube as shown in Figure S2.

An additional type K TC (TR1) is inserted into the reactant gas supply tube just below the orifice to measure the temperature of the gas before it enters the reactor. Another type K TC (TR13) records the temperature on the outside of the reactor lid. All TC positions along the height of the pyrolysis reactor *h*_R_ are given in Table S2 of the SI with the bottom end of the LM column set to *h*_R_=0 mm. TR10, TR11 and TR12 are located inside the argon inlet tube. A typical axial temperature profile is displayed in Figure S3 in the SI. Only the outlet temperature of the pyrolysis gas was measured by TR13, while the other temperatures, which are depicted in blue, correspond to the argon feed temperature, which increases towards the LM surface. The carrier gas was fed into the reactor in counterflow and the gas mixture leaving the reactor heated it up until it left the feed tube close to the LM surface. As the gas mixture leaving the reactor preheated the argon stream, the actual temperature profile of the gas leaving the reactor was higher than the argon temperatures depicted in blue.

The average temperature ${\bar{T}}_{LM}$ of TR1 to TR9, which are located within the LM filled part of the reactor, and its combined uncertainty are given in Table S3 for each of the carbon synthesis experiments. The reactor pressure is recorded upstream of the orifice (*p*_in_) and downstream of the gas outlet (*p*_out_). Argon (Ar 4.8) is fed into the upper reactor section through a central tube ending just above the LM surface. The addition of argon as a carrier gas ensures the continuous and complete removal of the fine carbon powder (micro‐ and nanoparticles, see second part^[29]^ for details) from the pyrolysis reactor. Diluting the product gas with argon is also intended to quench the pyrolysis reactions near the LM surface.

The solid carbon particles are separated from the mixture of argon and the pyrolysis product gas in particle filters after leaving the reactor. A fraction of the gas mixture then enters a gas chromatograph (GC) via a bypass line. Instead of the pyrolysis gas, also gas from the gas storage tank GST2 can be fed into the GC for analysis via another CH_4_ MFC located in a second bypass line. Table S3 lists the reaction parameters for the individual experiments. A typical time‐averaged temperature profile of the pyrolysis reactor is shown in Figure S3 in the SI. The methanation product gas compositions given in Table S4 in the SI were obtained by accounting for the time lag (see SI for details) between the methanation and the pyrolysis unit caused by the gas storage tank GST2, which connects the two process steps during the coupled process operation (see Figure 1).

*Analysis of Permanent Gases* A gas chromatograph (PerkinElmer ARNEL 6686 Model 4017) with four columns (HAYSEP N, HAYSEP T, molecular sieve 13X, molecular sieve 5 A) and a dual thermal conductivity detector operating with helium and nitrogen as carrier gases was used to analyse the permanent gases. The limit of quantification for all permanent gases analysed is given as 0.01 vol.% by the manufacturer. CH_4_, H_2_ and N_2_ were calibrated from 0.1 vol.% to 100 vol%. CO_2_, CO and C_2_H_*x*_ (with *x*=2, 4, 6) calibration was limited to low concentrations ranging from 0.1 vol% to 10 vol.%. Ar was calibrated from 25 vol% to 100 vol.%. Molar flow rates of the product gas were calculated from the known molar flow rate of argon (assuming ideal gas behavior) and the GC analyses. Details on the uncertainty analysis are given in the SI.

*Analysis of Carbon Yield* The mass of the particle filter bags was measured before and after each synthesis experiment (Kern 440–35 N scales, accuracy of ± 0.01 g). The mass difference was taken as the total mass of the product consisting of carbon species and tin particles. A CHN analyzer (LECO Truspec CHN Micro, Institute for Technical Chemistry (ITC) at KIT) was used to determine the mass fractions of C, H, N and the tin fraction was calculated by difference. This procedure was checked for two samples via ICP‐OES analysis (iCAP 7000 Series ‐ Thermo Scientific, Institute for Applied Materials – Applied Materials Physics (IAM‐AWP)), as given in table S7 in the SI. Given carbon yields refer to a tin‐free product. Details on the uncertainty analysis are given in the SI.

*Modelling* Equilibrium compositions shown in Figure S6 in the SI and Figure 6 were obtained from the Software Aspen Plus® V10 by minimizing the Gibbs free energy in an RGibbs reactor. In addition to solid carbon the following gas phase species were considered: CH_4_, H_2_, C_2_H_6_, C_2_H_4_, C_2_H_2_, N_2_, O_2_, CO_2_, CO, H_2_O, CH_2_O, CH_3_OH. The Cantera^[47]^ based model described in detail elsewhere^[48]^ was used to obtain the spatial profiles of C_2_H_*x*_ (*x*=2, 4, 6) mole fractions depicted in Figure 7.

## 3 Results and Discussion

### 3.1 Methanation of Carbon Dioxide

#### 3.1.1 Variation of Stoichiometry

**Figure 2:**
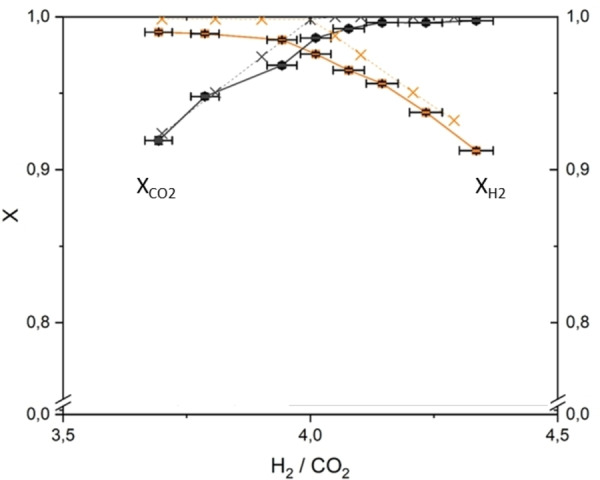
Results from hydrogen and CO_2_ conversion (continuous lines) as function of stoichiometry against thermodynamics (dotted lines). A setpoint for the plotted variation of stoichiometry was applied as follows: *T*_set_=320 °C; GHSV_total_ = 36 s^−1^; p=8,75 bar(a).

In the first set of experiments, performed independently of methane pyrolysis, it was determined what reactor temperature is the minimum in both stages for combining good conversion in terms of catalyst activity and selectivity due to thermodynamics. Thereby, the stoichiometry was changed around the ideal value of 4 to look at hydrogen and CO_2_ conversion. From Figure 2 it becomes clear that both conversions overlap but are slightly less than the thermodynamic value near 1. Such close approximation to full conversion and high selectivity is feasible from thermodynamics with the intermediate condensation of product water after stage 1 at 5 °C. Selectivity to methane is nevertheless near 100 % since there is no deviation between hydrogen and CO_2_ conversion also verified by the analysis of CO concentration. With slightly over stoichiometric hydrogen such as H_2_/CO_2_=4.2, the conversion of CO_2_ approaches maximum, eliminating CO formation at the same time.

#### 3.1.2 Addition of Impurities

Within the next set of experiments, also run independently from pyrolysis, the oxygen tolerance, a possible impurity in the DAC process and a stress parameter for the methanation catalyst with regard to the required Ni(0) active site, was evaluated (see Table S1 in the SI). The oxygen concentrations were applied to only reactor 1 for 45 h at each setpoint. The value of oxygen was increased from experiment to experiment and between the levels a standard operation point without oxygen was repeated. The standard point was selected in a way that methane selectivity is very high but full conversion is not reached to indicate even slight catalyst degradation. Within the individual oxygen experiments a certain higher amount of water product was found, and the CO_2_ conversion level decreased slightly due to the occurrence of the side reaction of hydrogen oxidation.

**Figure 3:**
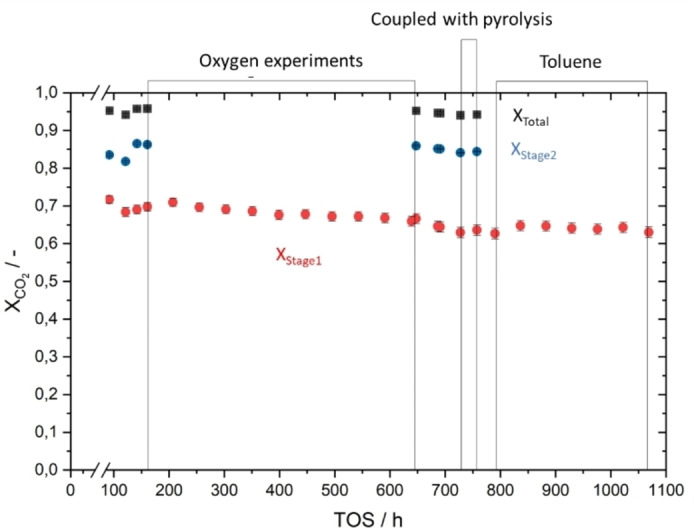
Results from the entire experimental campaign: overall CO_2_ conversion and conversion levels in the first and second stage (equilibrium conversion in first and second stage is close to 100 %). Data is provided for the experiments with artificial impurities oxygen and toluene and the reference points in between. Indicated is also the coupling phase with DAC and the pyrolysis unit of KIT (without data). The reference setpoint for the plotted activity measurement was applied as follows: *T*_set_=300 °C; GHSV=7.05 s^−1^; p=8,75 bar(a); H_2_/CO_2_=4.3.

From Figure 3 it becomes clear that the catalyst activity operated at standard point between the individual experiments of different oxygen levels did not change its trend. Thus, we conclude that oxygen impurity only harms the methane selectivity when oxygen is in the gas stream. However, the water formation does not take influence on catalyst deactivation up to levels of 1 vol.% O_2_.

A similar set of experiments was performed also with toluene, which is a possible candidate for biogas impurities, for the case that DAC is replaced by other CO_2_ sources. For the toluene levels see also Table S1 in the SI. No influence on methanation was found also in these experiments, neither regarding catalyst activity nor any increase of pressure drop as an indicator for coking.

#### 3.1.3 Operation with CO_2_ from DAC

From Figure 3 it can also be seen that in time coupling with pyrolysis, where DAC based CO_2_ was used, no considerable decline can be found from the data before or after. Nevertheless, the time of application of DAC based gas was not long (45 h only). Here, it might be necessary to extend the experimental time further. Figure S4 (top) in the SI shows a detailed analysis of the inert gas, CO_2,_ and methane concentration in the product gas over the experiment time during coupled operation when the methanation unit was run with CO_2_ surplus. It can be detected that the gas concentrations fluctuated, which was proven to originate from variations of the gas composition provided by the DAC unit. For comparison, Figure S4 (bottom) depicts the stable CO_2_ and CH_4_ concentrations in the methanation product gas when all reactant gases are taken from gas bottles instead.

Due to the variations in the DAC gas composition during coupled operation, the CO_2_ concentration was not stable leading to slightly varying methane purity. Nevertheless, the conversion remained above 95 % and the reactor stages were able to tolerate this fluctuation quite effectively. CO was less than 0.2 % in the methanation product gas (MPG) of all coupled experiments. All in all, CO_2_ excess was found to result in more stable product gas composition and flow, which shows in the significantly lower combined uncertainties given in Table S4 of the SI.

As no negative effects of CO or CO_2_ on the operability of the pyrolysis unit were noticed, a total of three coupled experiments (#2–4, see Table S3 and Table S4 in the SI) were run with CO_2_ excess and only one with H_2_ surplus (#1). The average gas volume fractions ${\bar{y}}_{i,MPG}$ of the respective MPG and the combined uncertainties, as obtained from IR and GC analysis according to the procedure given in the SI, are listed in Table S4. The difference to 100 % is given as ${\bar{y}}_{div,MPG}$ and consists mainly of N_2_ but also Ar. Samples taken from the gas storage tank (GST2) for GC analysis (after experiments #1, #2, #4) showed a small Ar peak in the chromatogram. As it was far beyond the calibrated concentration range of Ar the obtained volume fractions ${\bar{y}}_{Ar,GST2-GC}$ (2.4–3.4 %) can only be regarded as rough estimates. To get a better idea of the accuracy of those estimated values, we carried out a comparison of the volume fractions obtained by IR analysis of the MPG towards the end of an experiment and the volume fractions obtained by GC analysis of the GST2 gas mixture after the respective experiment. A comparison of ${\bar{y}}_{div,MPG}$ with ${\bar{y}}_{N2,GST2-GC}$ showed an overall large discrepancy, while ${\bar{y}}_{CH4,MPG}$ and ${\bar{y}}_{CH4,GST2-GC}$ were in good agreement. However, the sum of ${\bar{y}}_{Ar,GST2-GC}$ and ${\bar{y}}_{N2,GST2-GC}$ matched ${\bar{y}}_{div,MPG}$ a lot better (see Figure S5 in the SI), indicating that an actual volume fraction $y_{Ar,GST2}$ within the single figure percentage range is likely. GC analysis of the gas mixture in the GST2 did not detect C_2_H_*x*_ (*x*=2, 4, 6) byproducts.

#### 3.1.4 Post‐Mortem Catalyst Analysis

The catalyst was withdrawn from the reactors after 1200 h of operation. From physisorption a BET surface decline of average 24 % in reactor 1 and 18 % in reactor 2 was found. This indicates in our opinion the only slight correlation of the catalyst stability with conversion rates. Since both catalysts are not severely different in surface area decline and are not overheated in adiabatic mode as in classical reactors, this surface area effect seems more an aging effect of the support in the reaction mixture rather than a deactivation due to site reduction. Nevertheless, more detailed catalyst investigations could help to clarify this, such as pre‐ and post‐chemisorption or operando measurements.

### 3.2 Methane Pyrolysis

One of the objectives of the study is to explore the influence of the reactant gas mixture on the pyrolysis performance at various reaction temperatures. Depending on the process conditions chosen for the methanation step, the reactant gas may contain several percent of H_2_ or CO_2_, as given in Table S4. As mentioned before, the minimum humidity of the MPG was estimated at about 0.1 vol.% based on the equilibrium vapor pressure of water and the temperature and pressure of the condensation unit. As the actual water content of the MPG could not be quantified analytically and might have been higher than the minimum content, H_2_O is not listed as an individual component in Table S4 but is included in ${\bar{y}}_{div,MPG}$ . The following subsection 3.2.1 presents the effect of pyrolysis temperature on methane conversion *X*_CH4_, hydrogen yield *Y*_H2,CH4_ and carbon yield *Y*_C,CH4_. Details about the calculation of *X*_CH4_, *Y*_H2,CH4_,*Y*_C,CH4_ and their respective uncertainties are given in section 3 of the SI. Results from experiments with bottled gas as well as with MPG mixtures from the coupled operation are included. The effect of impurities contained in the MPG mixtures is analyzed by comparison with results obtained with pure CH_4_ and CH_4_‐N_2_ mixtures from bottled gas. Subsection 3.2.2 discusses the influence of methane concentration in the pyrolysis feed gas (PFG) at 1050 °C. Finally, the effects caused by the addition of argon and the continuous removal of carbon from the upper reactor section are presented in subsection 3.2.3.

#### 3.2.1 Effect of CO_2_ and H_2_ Impurities

**Figure 4:**
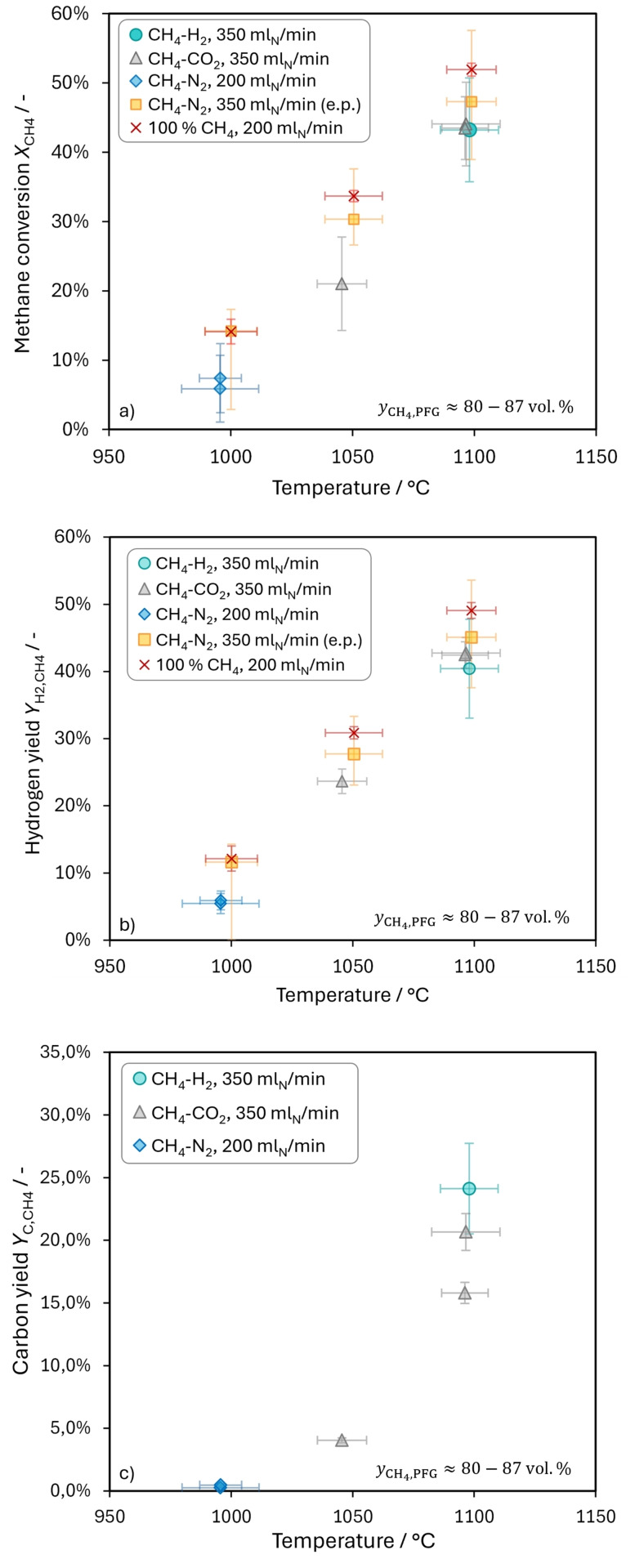
Effect of the average tin temperature on methane conversion *X*_CH4_ (a), hydrogen yield *Y*_H2,CH4_ (b) and carbon yield *Y*_C,CH4_ (c) with and without H_2_ or CO_2_ as impurities in the methane pyrolysis feed gas (PFG). Data presented in a) and b) as red crosses originates from a previous study^[46]^ and was reevaluated and extrapolated (e.p.) to obtain the data shown as yellow squares.

Figure 4 a and Figure 4 b display the effect of rising pyrolysis temperatures on methane conversion *X*_CH4_ and hydrogen yield *Y*_H2,CH4_ respectively. As reported in earlier studies,[^16^, ^18^, ^46^] *X*_CH4_ and *Y*_H2,CH4_ increase at higher temperatures. They remain far from equilibrium conversion and yield, though, which are close to unity for reaction temperatures of 1000 °C and above (see Figure S6 in the SI). Comparing the results obtained during the coupled process operation at 1050 °C and 1100 °C to methane conversions and hydrogen yields from a previous study with pure methane,^[46]^ reduced values of *X*_CH4_ and *Y*_H2,CH4_ are observed.

Between the PFG mixture containing mainly H_2_ as an impurity and the one containing mainly CO_2_, no difference can be noticed at 1100 °C, though. Regarding the uncertainties depicted for the conversions obtained previously,^[46]^ it must be noted that they only represent the standard deviation of three measurements. In contrast, a thorough uncertainty analysis has been conducted for the results of the present study (see SI), considering more factors than just the standard deviation, and thus yielding higher uncertainties.

Further differences arise from the carbon accumulation on top of the tin surface, the lower reactant gas volume flow, and the higher methane concentration Geißler^[46]^ used. To compensate the latter two differences, data from the previous study was reevaluated and extrapolated (e.p.) to estimate methane conversions and hydrogen yields that could be expected for an 85 : 15 CH_4_:N_2_ mixture when 350 ml_N_/min are fed into the bubble column reactor. Several types of carbon have been shown to catalyze the thermal decomposition of methane.[^49^, ^50^] The potential catalytic effect of the carbon layer is represented in the uncertainties of the e.p. values displayed in Figure 4 a and 4 b. Details on how the e.p. values and the respective uncertainties were calculated are given in the SI.

When *X*_CH4_ and *Y*_H2,CH4_ of the coupled operation are compared to the e.p. values and uncertainties are considered, negative effects of H_2_ or CO_2_ on methane conversion or hydrogen yield cannot be identified unambiguously. While the average values reached during the coupled process operation are lower than the e.p. values, the uncertainty ranges overlap.

While the carbon yield *Y*_C,CH4_ is lower than the hydrogen yield, it also increases noticeably in the temperature range from 1000 °C to 1100. For the investigated temperature range, the temperature dependency of *Y*_C,CH4_ seems to follow an exponential trend, as shown in Figure 4 c. All carbon yields are far from the thermodynamic equilibrium yields calculated for temperatures of 1000 °C and above (see Figure S6 b in the SI). The carbon yields shown in Figure 4 c cannot be compared to previous experiments conducted by Geißler et al.[^16^, ^17^] or Hofberger et al.[^18^, ^51^] as their carbon products weren′t removed from the reactor between experiments. Instead, they accumulated and mixed making a quantification relating to single experimental conditions impossible. The attribution of specific carbon samples to individual experiments was realized for the first time as part of this study. It was achieved by adding argon as a carrier gas for the continuous and complete removal of carbon from the pyrolysis reactor.

At 1100 °C, a discrepancy in the carbon yields obtained with PFG containing CO_2_ can be observed. Regarding the gas phase analysis (Figure 4 a and b), the results of the two experiments are very similar, though, indicating good repeatability. We assume that the different carbon yields are a result of a temporal blockage of the single orifice caused by tin weeping during the experiment with a higher carbon yield (#2 in Table S5 in the SI). After applying a nitrogen flow for troubleshooting, the reactor went through a transient stage where the reactant gas is mixed with nitrogen to reach the target feed gas composition again. During the transient phase, carbon formation is already expected to take place. As will be discussed in the following section, intermediate methane concentrations result in higher carbon yields. The carbon yield during the transient operation might therefore be higher than during steady state operation. Carbon yields were calculated with an integral approach (see SI for details), considering both transient and steady‐state operation. GC analysis of the gas phase on the other hand was only done once target conditions were reached. This could explain why no difference is seen for *X*_CH4_ and *Y*_H2_, but *Y*_C_ differs for the two experiments with CO_2_ at 1100 °C. The lower carbon yield of about 16 % obtained with CO_2_ in the PFG during uninterrupted pyrolysis operation is therefore compared to the reactant gas mixture containing H_2_. In this comparison at 1100 °C, the carbon yield is lower for a PFG mixture with CO_2_ than for one with H_2_ as an impurity. This is probably due to additional carbon consuming reactions, e. g. carbon gasification with CO_2_ or H_2_O (Equation (4) and Equation  (5)). OH radicals, CO_2_ and H_2_O could attack either carbon already synthesized or important gas‐phase intermediaries such as acetylene,[^52^, ^53^] thus reducing the final carbon yield. Besides, gas phase reactions such as dry reforming and steam reforming of methane (Equation (6) and Equation (7)) might compete with pyrolysis reactions and thus lower the amount of carbon precursors. In all coupled experiments, also the one with H_2_ as an impurity, some humidity remained in the gas mixture after the drying step. During the experiments with CO_2_ as an impurity, the presence of CO_2_ could have resulted in further steam formation via the rWGS reaction (Equation (2)). The analysis of the equilibrium constants *K*_p_ of the mentioned reactions (Figure S6 c in the SI) shows that at 1000 °C and above all of them would contribute to the formation of CO at the expense of carbon formation. In fact, the yield *Y*_CO,CO2_ was more than 40 % at 1100 °C and about 20 % at 1050 °C (see Table S5 in the SI), when CO_2_ was present in the PFG as an impurity.

After the operation of the pyrolysis reactor with CO_2_ and H_2_O containing reactant gas, no signs of tin oxidation were observed. Therefore, the overall reducing atmosphere either inhibited the formation of solid tin oxide or reversed it fast enough to prevent any noticeable oxidation.

#### 3.2.2 Effect of Methane Concentration

**Figure 5:**
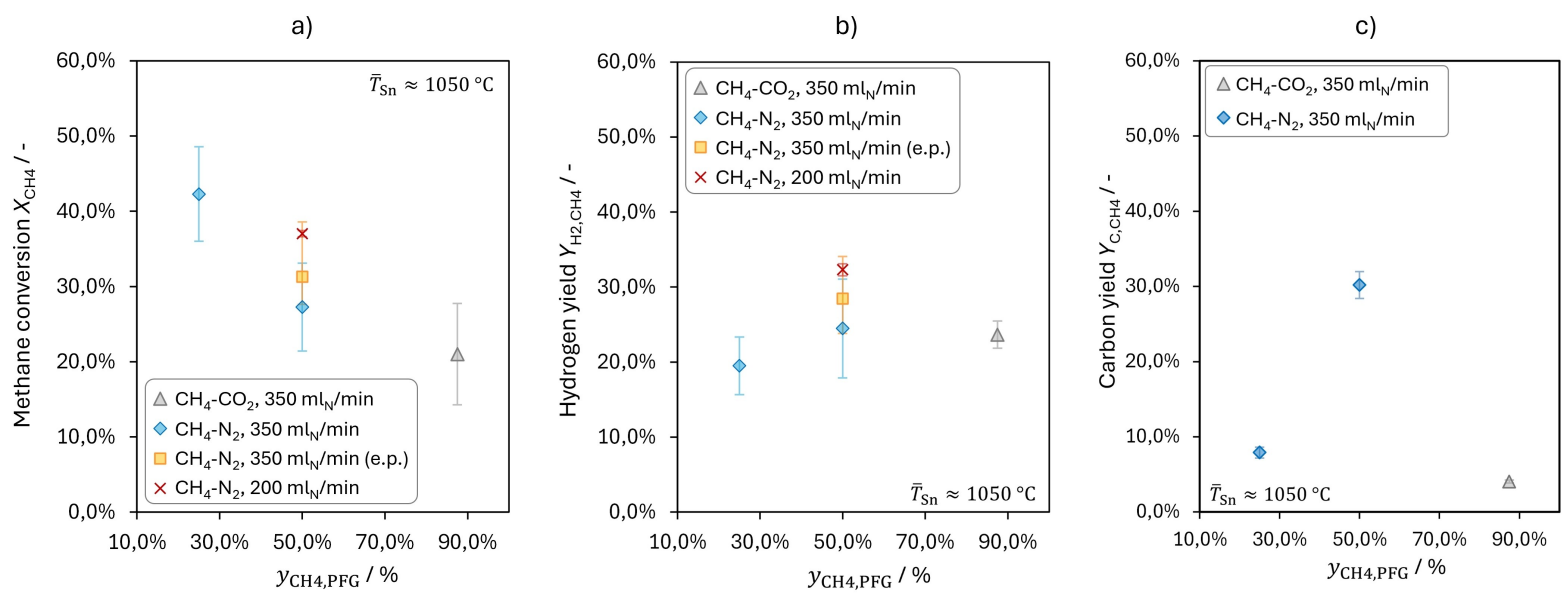
Effect of the methane volume fraction $y_{CH4,PFG}$ in the pyrolysis feed gas (PFG) on methane conversion *X*_CH4_ (a), hydrogen yield *Y*_H2,CH4_ (b) and carbon yield *Y*_C,CH4_ (c). Data presented in a) and b) as red crosses originates from a previous study^[46]^ and was reevaluated and extrapolated (e.p.) to obtain the data shown as yellow squares.

For a pyrolysis temperature of 1050 °C, the dilution of methane resulted in higher methane conversions, as depicted in Figure 5 a. This agrees with Geißler′s^[46]^ observation that methane conversions increased slightly when the methane volume fraction in the reactant gas was reduced.

However, he reported an absolute increase of only about 10 % over the range of 100 vol.% to 10 vol.% CH_4_ in the PFG.^[46]^ In contrast, Figure 5 a shows that *X*_CH4_ rises by more than 20 % when *y*_CH4,PFG_ is lowered from 87 vol.% to 25 vol.%. Reducing the methane concentration should result in slower reaction rates according to first order kinetics, e. g., the one suggested by Arutyunov and Vedeneev^[54]^ for the thermal decomposition of methane. On the other hand, dilution also lowers the gas volume increase due to the decomposition of one methane molecule into two hydrogen molecules (Equation (3)). Therefore, the bubbles remain smaller and rise slower when methane is diluted (in the relevant diameter range^[48]^). As a result, the residence time in the LM and in the upper gas filled part of the reactor increases. The latter needs to be considered as well when no quenching option is implemented. The observations of the present study, which support the findings of Geißler et al.,^[16]^ indicate that the increased residence time outweighs slower reaction rates, thus leading to higher methane conversions upon dilution. For our experiments, the conversion increase appears to result from effects in the LM as the pyrolysis product gas (PPG) was quenched with argon above the LM surface.

Hydrogen yields *Y*_H2,CH4_ obtained at 1050 °C and three different initial methane concentrations are depicted in Figure 5 b. In contrast to methane conversion, *Y*_H2,CH4_ does not show a trend. Reducing *y*_CH4,PFG_ from 87 vol.% to 50 vol.% results in no noticeable change of the hydrogen yield. With a further reduction of *y*_CH4,PFG_ to 25 vol.%, *Y*_H2,CH4_ seems to decrease. The uncertainty ranges for the yields obtained with 25 vol.% and 50 vol.% CH_4_ in the PFG overlap, though. A decrease of the hydrogen yield for low initial methane concentrations contrasts Gleißler′s^[46]^ findings.

For a pyrolysis temperature of 1175 °C, he observed increasing methane conversions and hydrogen yields upon methane dilution from 100 vol.% down to 10 vol.%. However, he did not test methane concentrations lower than 50 vol. % at 1050 °C, so the dilution effect might depend on pyrolysis temperature. Besides, the accumulated carbon layer on top of the LM might have affected his results in a different way than the gas quenching and continuous carbon removal of the present study.

The concentration dependent carbon yields displayed in Figure 5 c show a maximum for 50 vol.% CH_4_ in the PFG. For 25 vol.% CH_4_, hydrogen and carbon yields are significantly lower than methane conversion, indicating a low selectivity to the final pyrolysis products hydrogen (*S*_H2,CH4_ = 46.1± 11.4 %) and carbon. The carbon yield for the highest initial methane concentration was probably reduced by reactions of carbon or carbon precursors with CO_2_ and H_2_O as discussed in the previous section. Hydrogen inhibition effects^[55]^ might also be more noticeable for higher initial methane concentrations. For the same methane conversion, the partial pressure of H_2_ would be higher for a methane rich reactant gas, thus blocking more surface sites.

#### 3.2.3 Effect of Carrier Gas Addition

In contrast to previous pyrolysis experiments in bubble column reactors filled with liquid tin,[^16^, ^17^, ^18^, ^46^, ^51^] argon was fed into the pyrolysis reactor just above the LM surface with two objectives. Firstly, it continuously removed the fine carbon powder produced by the pyrolysis reactions from the reactor and transported it to a filter for later analysis. The successful removal of the carbon powder by argon flushing was verified by visual inspection of the reactor interior at the end of an experimental campaign once the tin column had cooled down and solidified. After previous experiments without argon addition, the upper part of the reactor was filled with compacted carbon powder. In contrast, after experiments with argon flushing hardly any carbon residues were found and the space above the solidified tin column was empty.

The second purpose of adding argon was to quench the pyrolysis reactions just above the LM surface to limit the carbon synthesis to the bubbles inside the tin column, which are considered close to isothermal (see Figure S3 in the SI). Without the addition of argon, the residence time in the upper section is longer than in the LM filled part^[16]^ (see Figure S7 in the SI) and temperatures are still high enough for reactions to continue after the gas leaves the LM. Comparing the spatial temperature profile obtained with argon addition (Figure S3 in the SI) to the ones previously published,[^46^, ^51^] there is hardly any difference observable. However, a much higher cooling rate was realized in the case of 9 L_N_ min^−1^ Ar addition by significantly speeding up the gas velocity, thus reducing the residence time of the gas mixture in the hot reactor zone. Based on model calculations (Figure S7 in the SI), the gas temperature above the LM surface drops below 500 °C within <0.8 s, while it takes 15.2 s without Ar addition.

Furthermore, the quenching effect of dilution was exploited when 9 L_N_ min^−1^ argon were added. An examination of the gas phase composition indicates that the dilution and the strong increase of the cooling rate suffice to successfully quench the pyrolysis product gas. The GC analyses from the experiments with argon addition above the LM surface show low amounts of ethylene and acetylene but no ethane. When no argon was fed into the top reactor section, Geißler et al.^[16]^ and Hofberger et al.^[18]^ also detected ethylene as a side product of methane pyrolysis in reactors filled with liquid tin. However, the detection of acetylene instead of ethane is a significant difference to these previous studies. This finding agrees with the following analysis of the temperature dependent equilibrium composition and kinetic modelling.

**Figure 6:**
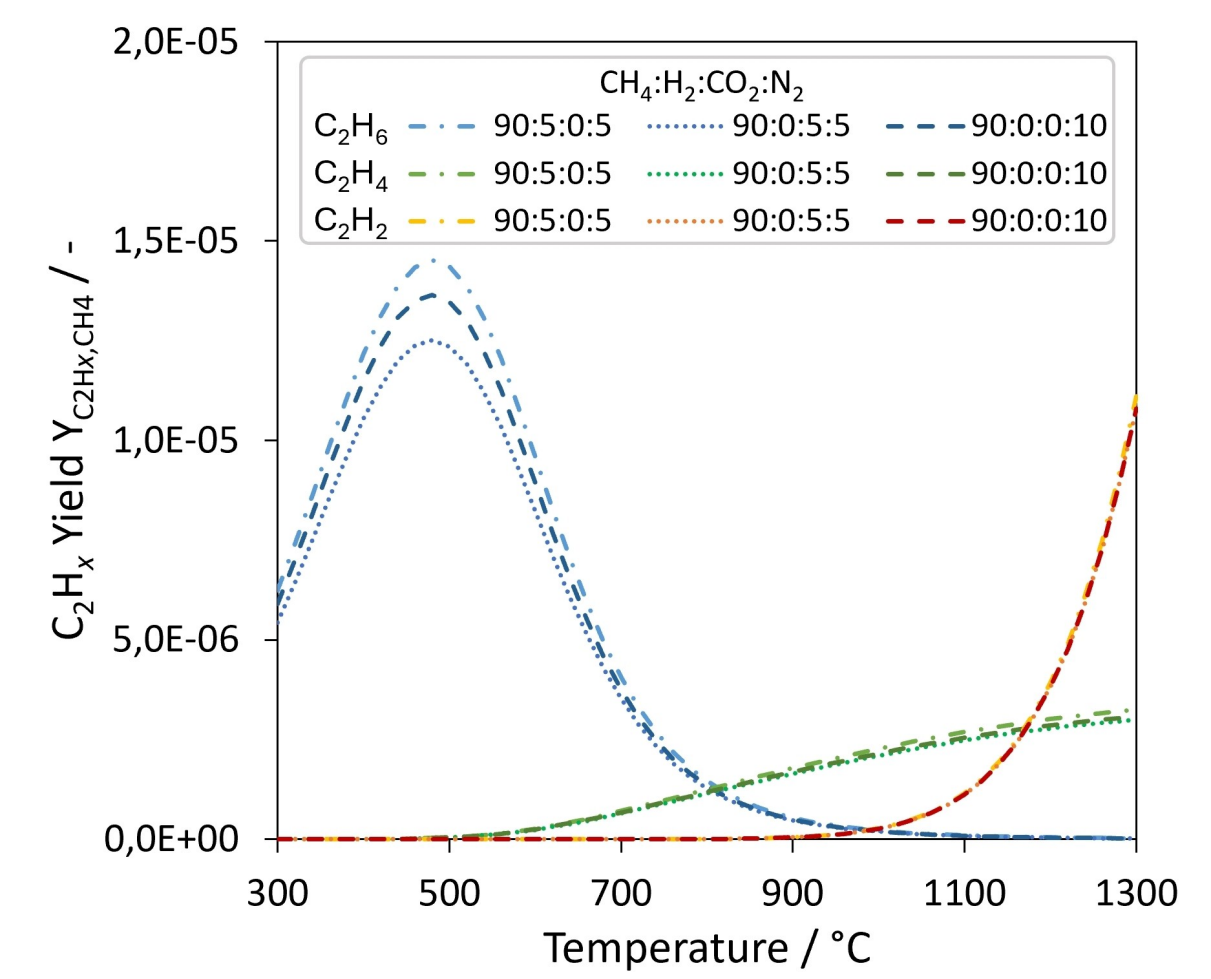
Thermodynamic equilibrium yields of C_2_H_*x*_ (with *x*=2, 4, 6) over the temperature range from 300 to 1300 °C for reactant mixtures containing either only CH_4_ and 10 % N_2_ or 5 % N_2_ mixed with 5 % of either CO_2_ or H_2_.

Figure 6 shows that for C_2_H_*x*_ (*x*=2,4,6) byproducts, at approximately 1000 °C the equilibrium composition of the gas phase shifts from containing ethane to containing acetylene. While lowering the temperature results in a sharp increase of ethane, higher temperatures lead to an exponential rise of the acetylene concentration. Ethylene experiences only a moderate increase. Based on the temperature of the liquid tin in the pyrolysis reactor and equilibrium considerations, acetylene and not ethane would be expected as a minor byproduct.

In a previous article,^[48]^ we introduced and validated a numerical model that, in agreement with experimental results,^[46]^ predicts ethane and ethylene as byproducts when the temperature gradient and gas residence time in the upper part of the reactor is considered. This model is now used to get a spatial resolution of the gas phase composition inside the optically non‐accessible pyrolysis reactor.

**Figure 7:**
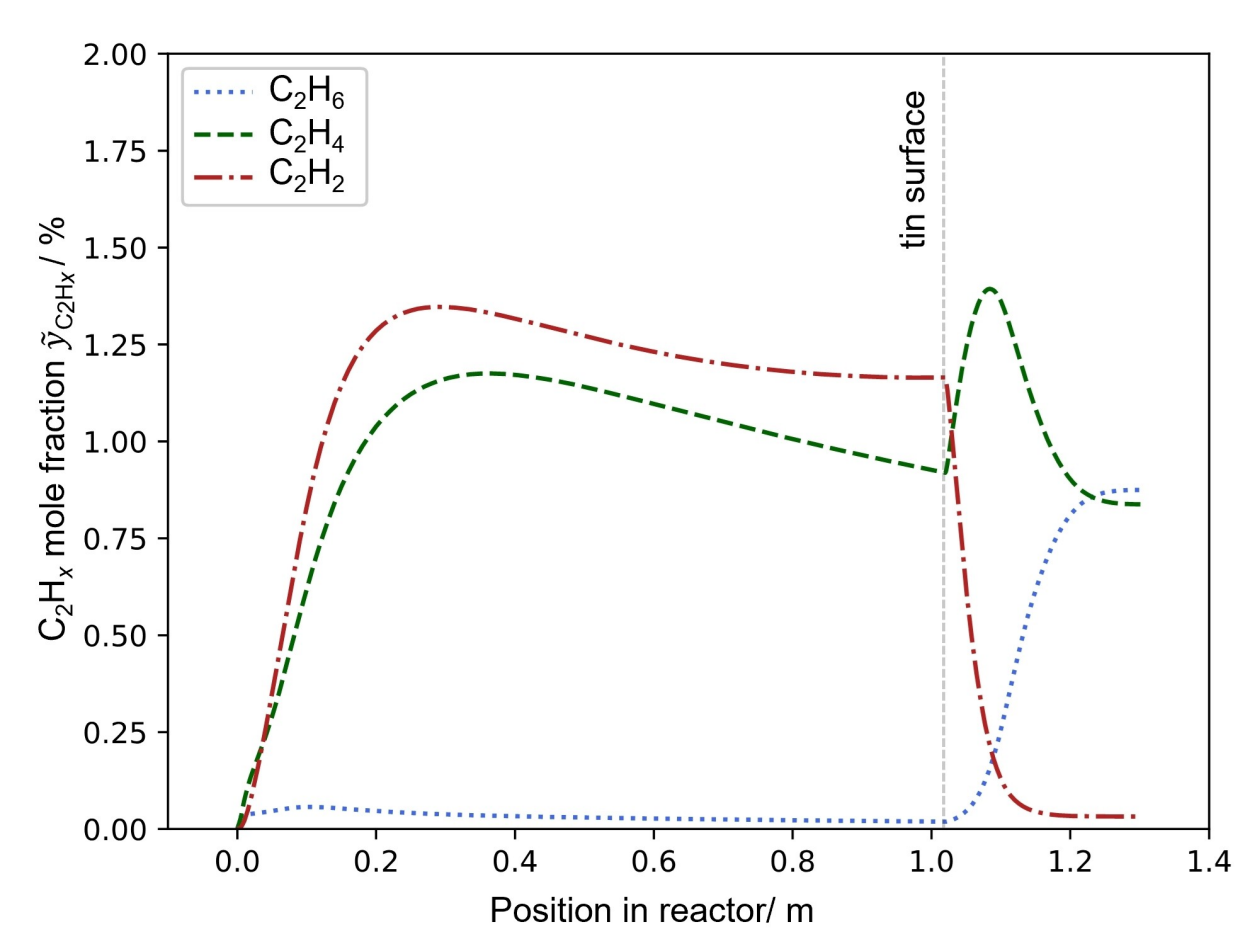
Modelled C_2_H_*x*_ (*x*=2, 4, 6) molar fractions along the length of the bubble column reactor showing C_2_H_4_ and C_2_H_2_ production inside the gas bubbles. Without quenching above the LM surface, the molar fraction of C_2_H_2_ drops while C_2_H_6_ increases during the slow cooling of the product gas. Model conditions: *T*_LM_=1100 °C, ${\dot{V}}_{reac}=350$ ml_N_ min^−1^, gas composition of experiment #1 (see Table S4 in the SI).

Figure 7 depicts the evolution of the molar fractions ${\tilde{y}}_{C2Hx}$ (with *x*=2, 4, 6) along the length of the reactor when no argon is added above the LM surface. The average gas composition of the coupled operation with H_2_ surplus (#1 in Table S4 in the SI) and an LM temperature of 1100 °C were chosen as input parameters for the model.

Acetylene and ethylene are present in small amounts in the LM filled lower part of the reactor after a short initiation period. The concentration of ethane on the other hand remains close to zero. Above the LM surface, the most striking change in the gas phase composition is the complete shift from acetylene to ethane caused by the slow cool down without Ar addition. This matches the experimental observations that were previously reported,[^16^, ^18^, ^46^] where no acetylene was detected in the product gas leaving the reactor. In the present study, the addition of argon above the LM surface was intended to restrict the pyrolysis reactions to the bubbles inside the LM. Successful quenching would be indicated by a gas composition, which matches the mixture at the LM surface. According to our model, acetylene and ethylene should then be present in small amounts, instead of ethane and ethylene. This is exactly what we found when argon was added as a carrier gas (see Table S6 in the SI for all pyrolysis product gas compositions).

Comparing the modelled acetylene molar fraction ${\tilde{y}}_{C2H2,m}$ of 1.16 % at the LM surface in Figure 7 to our experimental results ($y_{C2H2,exp}=$ 0.91± 0.40 vol.%) under the assumption of ideal gas behavior, there is good agreement between model and experiment. As already noted in our previous article,^[48]^ the model overpredicts ethylene concentrations (${\tilde{y}}_{C2H4,m}=0.89\;\%,\;y_{C2H4,exp}=0.40\;\pm 0.14\;\;vol\;\%)$ but a lower ethylene molar fraction compared to acetylene is found both in the experimental and the model results. They also agree regarding the absence of ethane. All in all, the byproduct analysis demonstrates the successful quenching of the product gas above the LM surface. This is an important foundation for the carbon characterization presented in part 2^[29]^ of this study as it limits the pyrolysis reactions to the close to isothermal part of the reactor. Previously, the reaction conditions in the upper reactor section were only poorly defined. Radial temperature profiles, the effect of the carbon layer and the degree of backmixing were unknown. By quenching the pyrolysis gas and continuously removing the carbon powder, these uncertainties in the carbon synthesis conditions can be reduced significantly.

A further effect of adding argon and continuously removing the carbon powder is the reduction of both methane conversion and hydrogen yield, as shown in Figure 4 a and Figure 4 b, respectively. At 1000 °C, a reactant gas flow of 200 ml_N_/min was fed into the reactor (blue diamonds), just as in previous experiments with pure methane (red crosses). The dilution of methane with nitrogen (8 : 2) cannot explain the reduced conversions and yields, as it should rather have the opposite effect (see previous section 3.2.2). Therefore, the addition of argon is seen as the cause for the lower methane conversions and hydrogen yields. This agrees with Palmer et al.’s^[25]^ observation that the addition of argon to the reactor head space lowered methane conversion due to the reduced residence time. They did not report any changes in the byproduct distribution, though. As they added a smaller argon stream, the carbon powder still collected on the surface of the molten metal and might have affected the reactions above the LM surface.

Figure S8 in the SI shows data from previous experiments without carbon removal.^[46]^ There is a noticeable increase over time for both *X*_CH4_ and *Y*_H2,CH4_ at 1000 °C, when the carbon powder is left to accumulate in the upper part of the reactor. This hints at a catalytic activity of the produced carbon. Other studies[^49^, ^50^] have also reported catalytic effects of carbon in the context of methane pyrolysis, which supports the assumption that the accumulated carbon layer catalyzed further pyrolysis reactions in the upper reactor section. With the addition of argon, the amount of potentially catalytic carbon is reduced to the particles in the gas stream and the cooling rate increases sharply. Both would be expected to reduce *X*_CH4_ and *Y*_H2,CH4_. The lower uncertainty ranges displayed in Figure 4 a and Figure 4 b for the extrapolated (e.p.) values (yellow squares), comprise the potential effect of carbon removal as explained in the SI. When the lack of a carbon layer is taken into account, *X*_CH4_ and *Y*_H2,CH4_ obtained with argon addition lie within the range that would be expected for continuous carbon removal.

### 3.3 Coupled Process Operation

The overarching aim of the coupled CCU process is to produce carbon from atmospheric CO_2_. The highest total carbon yield from CO_2_ could probably be achieved by combining methanation with H_2_ surplus with pyrolysis at high temperatures. Additionally, the upper gas filled section of the pyrolysis reactor could intentionally be used to increase the carbon yield. As can be seen from Table S4 in the SI, an over stoichiometric amount of H_2_ results in almost complete (99.915 %) conversion of CO_2_ into methane. The carbon yield of the entire process is then determined by the carbon yield of the pyrolysis step, which was higher for a gas mixture containing H_2_ than for a reactant gas containing CO_2_. In this study, the overall carbon yield achieved with H_2_ surplus and a pyrolysis temperature of 1100 °C is 24.1 %. However, there is a strong influence of the pyrolysis temperature on the carbon yield, as shown in Figure 4 c. In previous studies,[^16^, ^46^] hydrogen yields could be increased up to 78 % in a comparable reactor by setting the pyrolysis temperature to 1175 °C. The carbon yield is expected to increase along with the hydrogen yield, as we demonstrated in this study for the temperature range between 1000 °C and 1100 °C (Figure 4 b and Figure 4 c). A significant further increase of *Y*_C,CO2_ should therefore be achievable at higher pyrolysis temperatures. When no isothermal carbon synthesis conditions are required, another optimization option to further increase the carbon yield would be to intentionally include the upper gas filled reactor part as a carbon formation zone. The carbon powder could then be removed batchwise to make use of the catalytic activity of the carbon.

## 4 Conclusions

For the first time, a carbon capture and utilization (CCU) process that combines direct air capture (DAC), catalytic methanation and methane pyrolysis to convert CO_2_ into solid carbon materials was demonstrated in coupled operation. An overall yield *Y*_C,CO2_ = 24.1 % of carbon powder from atmospheric CO_2_ was obtained during the coupled operation of the process steps.

Regarding the methanation step, stable operation with either a surplus of CO_2_ or H_2_ in the reactant mixture was demonstrated. Due to fluctuations in the feed CO_2_ concentration, variations in the methanation product gas were observed. While for both modes of operation the CH_4_ content of the product gas was high (>80 vol. %), CO_2_ methanation with CO_2_ surplus yielded a more stable product gas composition. The synthesized methane contained up to 5.6 vol% CO_2_, 0.02 vol% CO and about 1 vol% H_2_. An average H_2_ concentration of about 10 vol% remained in the product gas mixture when the methanation was operated with a surplus of H_2_, while CO and CO_2_ were almost eliminated as side components. In addition to CO, CO_2_ and H_2_, up to 8 % of other gases (N_2_ and Ar) were detected in the gas mixture after the methanation step. Except for the observed fluctuations of the product gas composition, the utilization of CO_2_ from the DAC unit showed no adverse effects on the operability of the methanation unit. Toluene and oxygen are both likely impurities of CO_2_ provided either from a biogas plant or a DAC unit, respectively. In separate tests, no negative effects of those two impurities on methanation performance and catalyst stability were observed.

The methane pyrolysis unit was operated with both types of methane gas mixtures obtained from the catalytic methanation of atmospheric CO_2_. The operability of the pyrolysis reactor was not affected by the presence of CO, CO_2_, H_2_, small amounts of H_2_O or other impurities. No signs of tin oxidation were observed after conducting experiments with oxygen containing components. With either gas mixture comparable methane conversions *X*_CH4_ and hydrogen yields *Y*_H2,CH4_ were obtained. Regarding the solid carbon yield, the mixture containing mainly H_2_ as a side component resulted in higher *Y*_C,CH4_, though. The reduced carbon yield obtained with reactant gas containing CO_2_ is caused by reactions consuming carbon or intermediate products, which resulted in noticeable CO formation. The solid carbon product was successfully removed from the pyrolysis reactor via continuous pneumatic conveying. Thus, the carbon samples could be matched to their respective synthesis conditions and temperature dependent carbon yields of methane pyrolysis in a liquid metal filled bubble column reactor were reported for the first time. *Y*_C,CH4_ was shown to increase from about 0.5 % at 1000 °C to about 24.1 % at 1100 °C. This article is the first of two parts. Details about the synthesized solid carbon products and possible applications are presented in the second part.

The inert carrier gas used to remove the carbon powder was shown to act as a quenching agent. The quenching of the pyrolysis product gas resulted in the detection of acetylene as a byproduct in contrast to ethane detection in previous experiments without quenching. The presence of acetylene in the pyrolysis product gas is in good agreement with a thermodynamic analysis and kinetic modelling.

## Conflict of Interests

The authors declare no conflict of interest.

5

## Supporting information

As a service to our authors and readers, this journal provides supporting information supplied by the authors. Such materials are peer reviewed and may be re‐organized for online delivery, but are not copy‐edited or typeset. Technical support issues arising from supporting information (other than missing files) should be addressed to the authors.

Supporting Information

## Data Availability

The data that support the findings of this study are available in the supplementary material of this article.
