# Supplementary material for: A Carbon Capture and Utilization Process for the Production of Solid Carbon Materials from Atmospheric CO2 – Part 1: Process Performance
Source: ChemSusChem. 2024 Nov 12;18(5):e202401779. doi: 10.1002/cssc.202401779 (PMC11874706; doi:10.1002/cssc.202401779)
Supplement: Supplementary file 1 — Supporting Information [file CSSC-18-e202401779-s001.pdf]

ChemSusChem

Supporting Information

A Carbon Capture and Utilization Process for the Production of Solid Carbon Materials from Atmospheric CO_2 – Part 1: Process Performance

Neele Uhlenbruck,* Peter Pfeifer, Benjamin Dietrich, Christoph M. Hofberger, Ralf Krumholz, Antonio Saxler, Linus Schulz, Leonid Stoppel, and Thomas Wetzel

A Carbon Capture and Utilization Process for the Production of Solid Carbon Materials from Atmospheric CO_2 – Part 1: Process Performance

Supplementary Information

Neele Uhlenbruck*[a], Peter Pfeifer [b], Benjamin Dietrich[c], Christoph Hofberger[a], Ralf Krumholz[a], Antonio Saxler[b], Linus Schulz[b], Leonid Stoppel[a], Thomas Wetzel[c]

[a] N. Uhlenbruck, C. M. Hofberger, R. Krumholz, Dr. L. Stoppel
Institute for Thermal Energy Technology and Safety
Karlsruhe Institute of Technology
Hermann-von-Helmholtz-Platz 1, 76344 Eggenstein-Leopoldshafen
E-mail: neele.uhlenbruck@kit.edu

[b] A. Saxler, Dr. L. Schulz, Prof. Dr. P. Pfeifer
INERATEC GmbH
Siemensallee 84, 76187 Karlsruhe

[c] Dr. B. Dietrich, Prof. Dr. T. Wetzel
Institute of Thermal Process Engineering
Karlsruhe Institute of Technology
Kaiserstr. 12, 76131 Karlsruhe

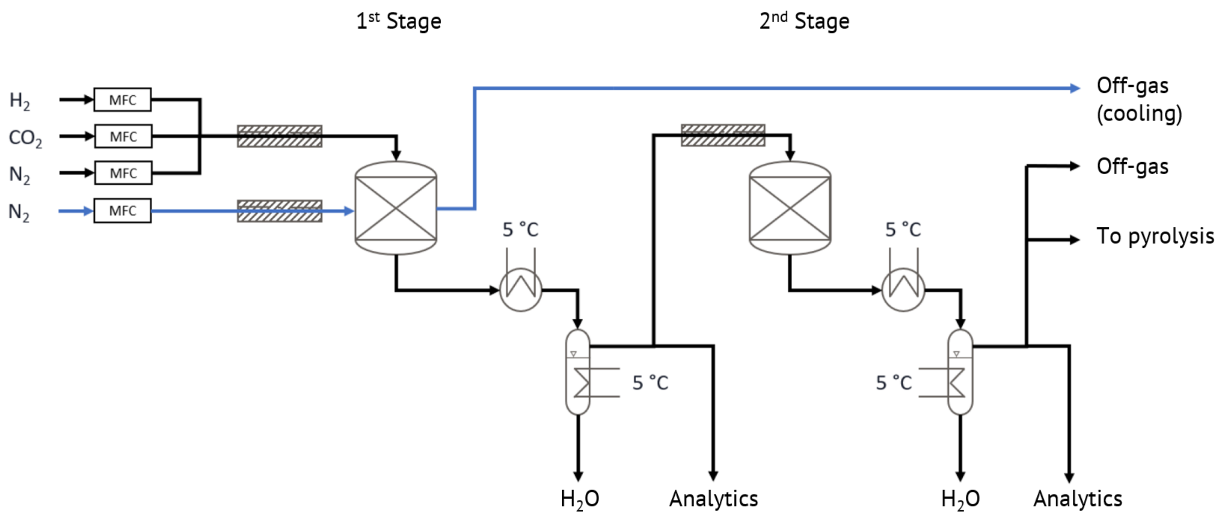

Figure S1. Process flow diagram of INERATEC's plant inside the NECOC process scheme to produce a high-quality methane gas stream for subsequent pyrolysis.

Table S1. Oxygen levels and toluene levels which have been applied to the first reactor.

O_2 ppm	Toluene Ppm
14,0	0,5
48,5	1,0
100,0	2,5
173,0	5,0
346,0	10,0
692,5	20,0
1385,0	-
2597,0	-
5020,5	-
10041,5	-

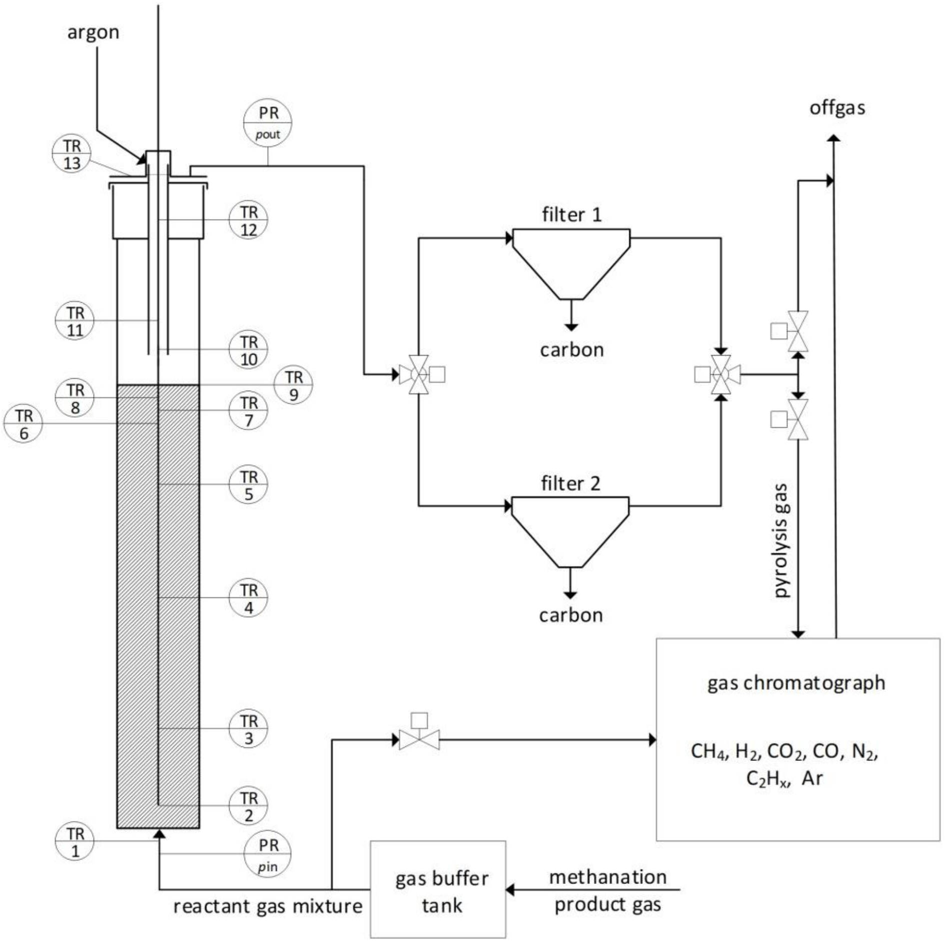

Figure S2. Simplified process flow diagram of the pyrolysis facility.

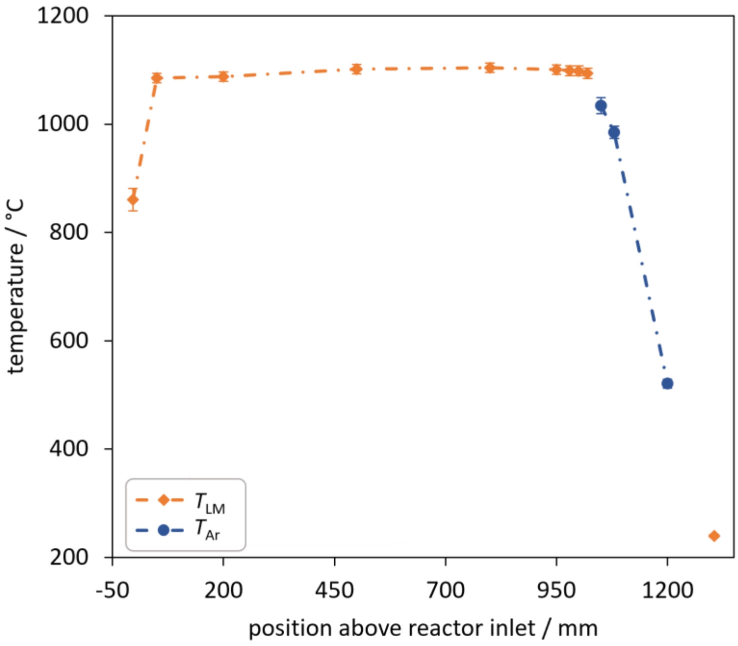

Figure S3. Temperature profiles along the length of the reactor when 9 L_N min^{-1} argon is added just above the liquid metal (LM) surface

Table S2. Vertical positions h_R of thermocouples (TC) inside the pyrolysis reactor

TC	h_R / mm	TC	h_R / mm	TC	h_R / mm	TC	h_R / mm
TR1	-4	TR5	800	TR9	1020	TR13	1310
TR2	50	TR6	950	TR10	1050		
TR3	200	TR7	980	TR11	1080		
TR4	500	TR8	1000	TR12	1200		

Table S3. Pyrolysis parameters. Experiments #1-#4 were run in coupled process operation, experiments #5-#8 were run as individual pyrolysis experiments with bottled gas.

Experiment	$\bar{T}_{LM}$ / °C	Reactant mixture	$\bar{p}_{in}$ / bar(a)	$\bar{p}_{out}$ / bar(a)	$\dot{V}_{reac}$ / ml$_N$ min^{-1}	$\dot{V}_{Ar}$ / l$_N$ min^{-1}
#1	1098.0 ± 11.9	see table S4	1.779 ± 0.062	1.015 ± 0.030	364.0 ± 25.6	9.000 ± 0.047
#2	1096.6 ± 14.0	see table S4	1.831 ± 0.186	1.083 ± 0.021	354.9 ± 6.9	9.000 ± 0.047
#3	1096.2 ± 9.6	see table S4	1.840 ± 0.050	1.089 ± 0.011	358.0 ± 10.1	9.000 ± 0.047
#4	1045.6 ± 10.1	see table S4	1.815 ± 0.011	1.085 ± 0.009	353.5 ± 5.4	9.000 ± 0.047
#5	1048.2 ± 9.5	25:75 CH_4:N_2	1.752 ± 0.004	1.075 ± 0.008	350.0 ± 1.8	9.000 ± 0.046
#6	1048.5 ± 9.4	50:50 CH_4:N_2	1.770 ± 0.052	1.075 ± 0.008	350.0 ± 1.9	9.000 ± 0.046
#7	995.6 ± 15.8	80:20 CH_4:N_2	1.729 ± 0.005	1.084 ± 0.008	200.0 ± 1.0	12.000 ± 0.06
#8	995.7 ± 8.6	80:20 CH_4:N_2	1.728 ± 0.004	1.083 ± 0.008	200.0 ± 1.0	12.000 ± 0.06

Table S4. Averaged volume fractions of the methanation product gas (MPG) during the coupled operation of the CCU process. Experiments #1-4 refer to the same experimental runs #1-#4 in table S3, which were done in coupled process operation.

Experiment	$\bar{y}_{CH4,MPG}$/%	$\bar{y}_{H2,MPG}$/%	$\bar{y}_{CO2,MPG}$/%	$\bar{y}_{CO,MPG}$/%	$\bar{y}_{div,MPG}$/%
#1	82.46 ± 6.85	10.07 ± 5.77	0.06 ± 0.16	0.01 ± 0.08	7.40 ± 12.86
#2	87.01 ± 0.29	1.18 ± 0.22	4.31 ± 0.59	0.01 ± 0.08	7.49 ± 1.21
#3	86.85 ± 0.34	1.18 ± 0.29	4.03 ± 0.81	0.01 ± 0.08	7.93 ± 1.55
#4	87.49 ± 0.62	0.97 ± 0.35	5.56 ± 1.56	0.02 ± 0.09	5.96 ± 2.62

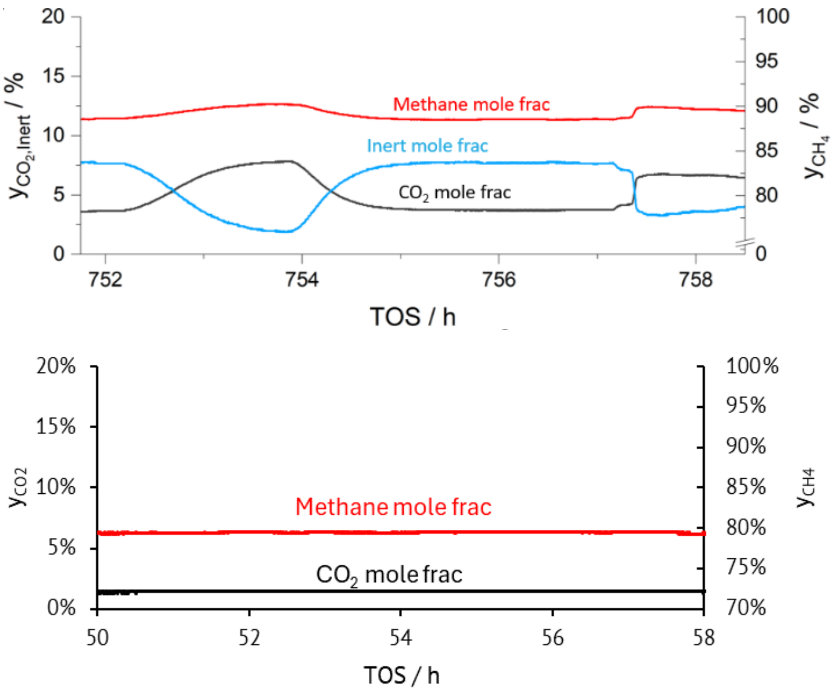

Figure S4. Top: CH_4, CO_2 and inert gas concentrations (the latter two found as reason for fluctuation of conversion) in the methanation product gas is plotted over the experiment time for the interconnected operation of DAC, methanation and pyrolysis. CO_2-surplus with setpoint as follows: T_{set}=340°C; GHSV=4 s^{-1}; p=9,2 bar(a); H_2/CO_2=3.82. Bottom: Stable gas concentrations during operation with bottle gases at reference point T_{set}=300°C; GHSV=7.05 s^{-1}; p=8,75 bar(a); H_2/CO_2=4.3 (deviation to 100% by excess H_2)

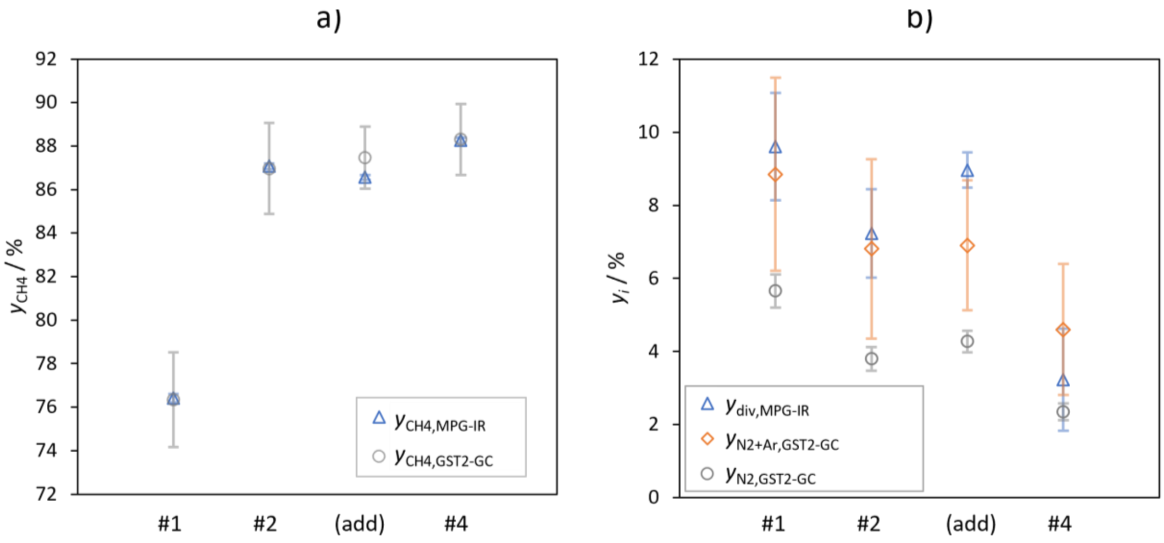

Figure S5. Comparison of the average volume fractions of methane and undetected gases (div) based on IR analysis of the methanation product gas (MPG) towards the end of the respective experiment with GC analysis of the gas mixture stored in the gas storage tank 2 (GST2). An additional (add) dataset is displayed, which is otherwise not included in the article.

Table S5. Pyrolysis process performance parameters with respective combined uncertainties u_C. Experiments #1-#4 correspond to the coupled operation of the CCU process.

Experiment	X_{CH4}	$Y_{H2,CH4}$	$S_{H2,CH4}$	X_{CO2}	$Y_{CO,CO2}$	$S_{CO,CO2}$	$Y_{C,CH4}$
#1 – 1100°C, CH_4-H_2	43.2 %	40.4 %	93.5 %	-	-	-	24.1 %
	± 7.5 %	± 7.4 %	± 28.7 %				± 3.6 %
#2 – 1100°C, CH_4-CO_2	44.1 %	42.8 %	97.0 %	46.0 %	43.1 %	93.6 %	20.7%
	± 6.0 %	± 2.3%	± 14.4 %	± 20.9 %	± 17.1 %	± 59.9 %	± 1.5%
#3 – 1100°C, CH_4-CO_2	43.5 %	42.5 %	97.6 %	46.6 %	44.4 %	95.2 %	15.8 %
	± 4.5 %	± 2.0 %	± 12.0 %	± 28.3 %	± 19.9 %	± 77.6 %	± 0.8 %
#4 – 1050°C, CH_4-CO_2	21.0 %	23.7 %	112.6 %	6.9 %	20.3%	293.6 %	4.0 %
	± 6.7 %	± 1.8 %	± 37.6 %	± 49.1 %	± 10.7 %	± 2132.8 %	± 0.2 %
#5 – 1050 °C, CH_4-N_2	42.3 %	19.5 %	46.1 %	-	-	-	7.9 %
	± 6.3 %	±3.9 %	±11.4 %				±0.7 %
#6– 1050 °C, CH_4-N_2	27.3 %	24.5 %	89.8 %	-	-	-	30.2 %
	± 5.8 %	± 6.6 %	± 30.9 %				± 1.8 %
#7– 1000 °C, CH_4-N_2	5.9 %	5.5 %	93.0 %	-	-	-	0.3 %
	± 4.8 %	± 1.5 %	± 80.5 %				± 0.1 %
#8– 1000 °C, CH_4-N_2	7.4 %	5.9 %	80.0 %	-	-	-	0.5 %
	± 5.0 %	± 1.4 %	± 57.0 %				± 0.1 %

Table S6. Average undiluted gas volume fractions $y_{i,PPG}$ of the pyrolysis product gas (PPG) with respective combined uncertainties u_C. C_2H_6 was not detected. Experiments #1-#4 correspond to the coupled operation of the CCU process.

Experiment	$y_{CH4,PPG}$ / %	$y_{H2,PPG}$ / %	$y_{C2H4,PPG}$ / %	$y_{C2H2,PPG}$ / %	$y_{N2,PPG}$ / %	$y_{CO2,PPG}$ / %	$y_{CO,PPG}$ / %
#1 – 1100°C, CH_4-H_2	36.95 ± 3.53	60.48 ± 5.17	0.40 ± 0.14	0.91 ± 0.40	1.26 ± 1.12	-	-
#2 – 1100°C, CH_4-CO_2	36.38 ± 3.24	56.52 ± 2.78	0.40 ± 0.14	0.80 ± 0.27	1.38 ± 0.56	1.74 ± 0.58	2.79 ± 1.04
#3 – 1100°C, CH_4-CO_2	36.81 ± 2.99	56.17 ± 2.51	0.40 ± 0.12	0.79 ± 0.24	1.53 ± 0.50	1.61 ± 0.78	2.69 ± 1.05
#4 – 1050°C, CH_4-CO_2	56.99 ± 19.27	34.94 ± 11.96	0.67 ± 0.33	0.65 ± 0.20	0.60 ± 1.16	4.26 ± 1.91	1.88 ± 0.83
#5 – 1050 °C, CH_4-N_2	14.63 ± 2.03	9.89 ± 2.32	0.00 ± 0.00	0.18 ± 0.22	75.30 ± 7.00	-	-
#6 – 1050 °C, CH_4-N_2	31.67 ± 3.15	21.30 ± 5.52	0.47 ± 0.15	0.47 ± 0.15	46.09 ± 5.82	-	-
#7 – 1000 °C, CH_4-N_2	76.25 ± 5.44	8.84 ± 2.55	0.00 ± 0.00	0.00 ± 0.00	14.75 ± 3.00	-	-
#8 – 1000 °C, CH_4-N_2	75.73 ± 5.55	9.68 ± 2.41	0.00 ± 0.00	0.00 ± 0.00	14.46 ± 3.01	-	-

3.2.1 Thermodynamic analysis

For a thermodynamic analysis, a 90:10 mixture of methane and nitrogen was compared to 90:5:5 mixtures, where half the nitrogen was replaced by H_2 or CO_2. The resulting equilibrium conversions of methane X_{CH4} and carbon yields Y_C as functions of temperature and reactant gas composition are shown in Figure S6 a and b, respectively. X_{CH4} is only slightly affected by H_2 and CO_2 in the low temperature range up to approx. 600 °C. The presence of CO_2 results in a shift of the pyrolysis equilibrium towards higher conversions as some H_2 is consumed by the reversed WGS reaction (Equation 2). The addition of H_2 to the initial reactant mixture has the opposite effect by shifting the pyrolysis equilibrium further towards CH_4 according to Le Chatelier's principle. At higher temperatures of 1000 °C and above, which cover the temperature range relevant for the pyrolysis system of this study, no differences regarding the equilibrium methane conversion are noted, though. While the effect of H_2 addition also has the same effect on the carbon yield $Y_{C,CH4}$, the presence of CO_2 in the reactant gas mixture shifts the equilibrium carbon yield in a different manner. In the lower temperature range, $Y_{C,CH4}$ also increases when CO_2 is added. This cannot be attributed to the reversed WGS reaction by itself, though, which does not yield any carbon. Instead, also the Boudouard reaction has to be considered for carbon formation from CO (Equation 4) in the low temperature range. This changes at about 700 °C, when the equilibrium is shifted towards the gasification of carbon. Figure S6 c displays the constants of equilibrium K_p for various reactions introduced before. Not only the Boudouard/gasification equilibrium changes sides, but all side reactions depicted, which were introduced before, undergo the shift from K_p <1 to K_p >1 in the relatively narrow temperature interval between 620 °C and 830 °C. In the presence of CO_2, the rWGS reaction provides H_2O as a further source for carbon gasification

according to Equation 5. With CO_2 and H_2O as gasifying agents, $Y_{C,CH4}$ is lowered at high temperatures when CO_2 is present in the reactant gas, compared to mixtures containing only N_2 or N_2 and H_2.

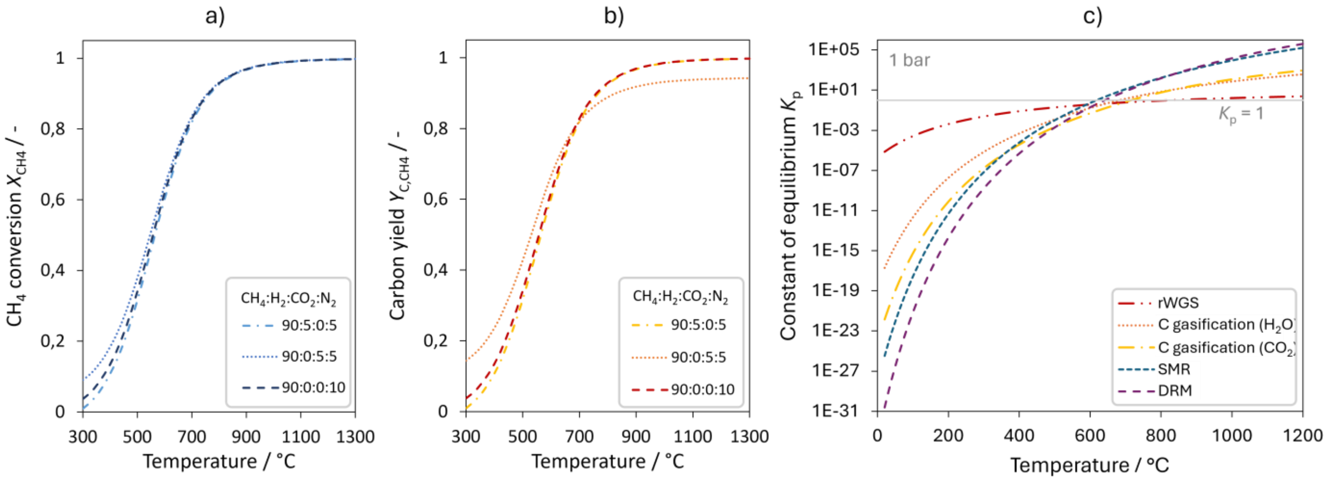

Figure S6. a) Thermodynamic equilibrium conversion of CH_4 X_{CH4} and b) carbon yield $Y_{C,CH4}$ as functions of pyrolysis temperature for reactant mixtures containing 90 % CH_4 in N_2 or a mix of 5 % N_2 and 5 % of either CO_2 or H_2. c) constants of equilibrium K_p for side reactions expected to occur when the pyrolysis feed gas contains CO_2.

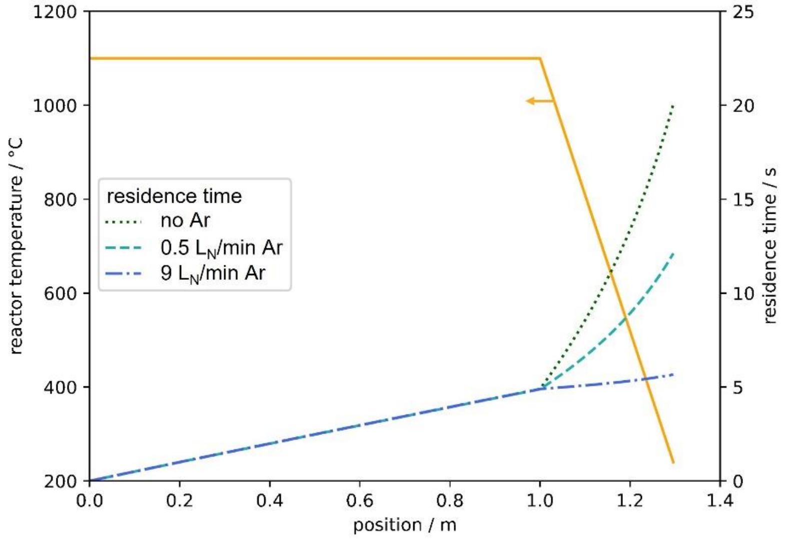

Figure S7. Modelled (according to our previous article [49]) residence time reduction as a result of argon addition above the liquid metal surface for an initial reactant gas flow of 350 ml_N min^{-1} (composition: 83.65 vol% CH_4, 13.03 vol% H_2, 3.22 vol% N_2, 0.1 vol% CO_2) and a liquid tin temperature of 1100 °C.

Table S7. CHN elemental analysis of the samples synthesized from atmospheric CO_2. The nitrogen blank value was 0.4-0.5 wt.%.

Sample	C in wt.%	H in wt.%	N in wt.%	metal in wt.% (by difference)	metal in wt.% (ICP-OES)
#1 – 1100°C, CH_4-H_2	19.2 ± 2.0	<0.2	0.60 ± 0.06	80.2	-
#2 – 1100°C, CH_4-CO_2	19.3 ± 0.1	<0.2	0.56 ± 0.07	80.1	-
#2 – 1100°C, CH_4-CO_2*	19.3 ± 1.1	<0.2	<0.2	80.7	-
#3 – 1100°C, CH_4-CO_2	15.0 ± 0.6	<0.2	0.54 ± 0.01	84.5	-
#4 – 1050°C, CH_4-CO_2	16.3 ± 0.4	<0.2	0.57 ± 0.05	83.1	-
#5 – 1050°C, CH_4-N_2	10.3 ± 0.8	<0.1	<0.1	89.7	-
#6 – 1050°C, CH_4-N_2	11.8± 0.6	<0.1	<0.1	88.2	-
#7 – 1000°C, CH_4-N_2	5.6 ± 1.1	0.98 ± 0.2	<0.1	94.2	96.7 ± 5.0
#8 – 1000°C, CH_4-N_2	5.7 ± 1.3	0.09 ± 0.2	<0.1	94.2	95.3 ± 5.0

*Repetition of analysis six weeks later with a nitrogen blank value of 0.1-0.2 wt.%

Estimation of methane conversions and hydrogen yields for 350 ml_N/min of CH_4-N_2 mixtures

Figures S8 to S10 depict data obtained from previous experiments [47], which has been reevaluated for the present study. During the previous pyrolysis experiments in a bubble column reactor filled with liquid tin, carbon formed as a powder and accumulated on top of the tin surface as the gas velocity was not high enough to carry it out of the reactor. Over the course of several days, the growing carbon layer resulted in an increase of methane conversion and hydrogen yield, as shown in Figure S8. Methane pyrolysis experiments at 1000 °C, 1050 °C and 1100 °C were conducted from day six onward. The preceding days were dedicated to experiments at higher and lower temperatures, which lie outside of the temperature range investigated in the present study. Estimates of X_{CH4} and Y_{H2} on the first day without carbon layer were obtained by linearly extrapolating the data from days six to thirteen.

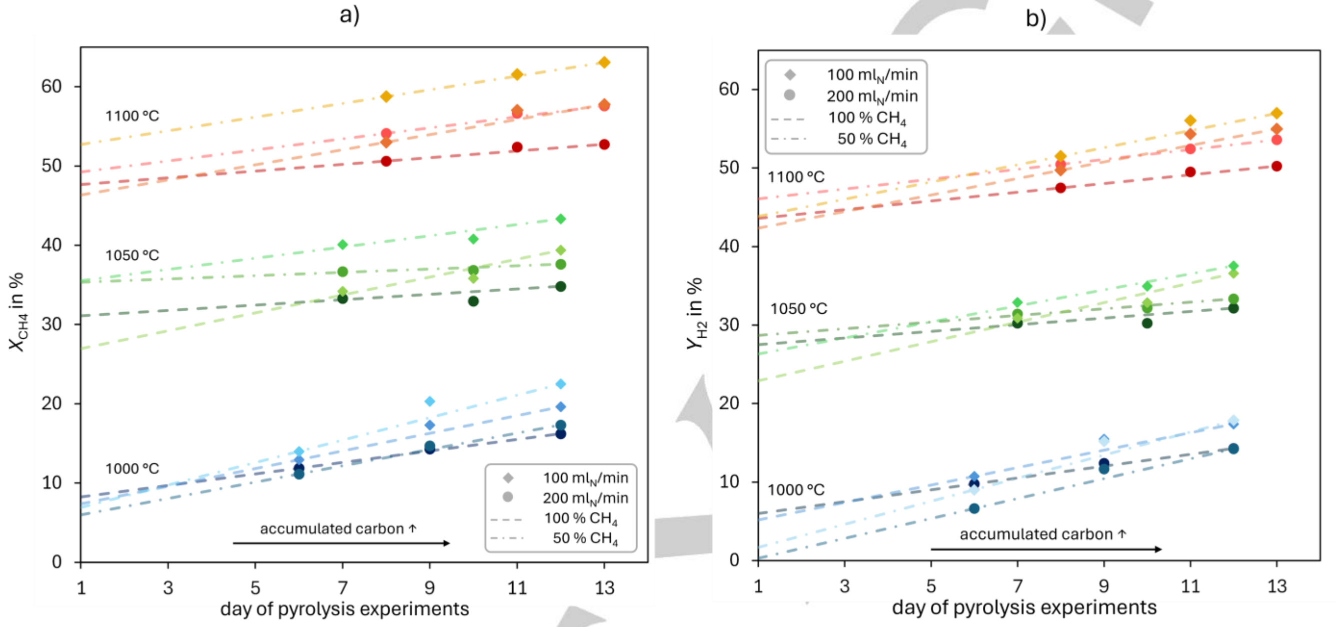

Figure S8. Influence of an accumulating carbon layer on top of the liquid metal surface on methane conversion and hydrogen yield. The depicted data (fille circles and diamonds) was obtained during previous experiments [47] and reevaluated for the present study. The lines show the linear extrapolations to day 1 without carbon layer.

The dilution of methane with N_2 (1:1) resulted in higher methane conversions and hydrogen yields, as shown in Figure S9 and Figure S10. Based on the results obtained for 100 vol.% and 50 vol.% CH_4 in the reactant gas mixture, X_{CH4} and Y_{H2} were estimated for a CH_4-N_2 mixture with 85 vol.% CH_4 by linear interpolation. The interpolation results are shown in Figure S9 and Figure S10.
Figure S9 and Figure S10 also show a strong dependency of X_{CH4} and Y_{H2} on the volume flow of the pyrolysis feed gas (PFG). To account for a potential further reduction of X_{CH4} and Y_{H2} when the volume flow is increased to 350 ml_N/min, the interpolated values (for a CH_4-N_2 mixture with 85 vol.% CH_4) were extrapolated with exponential fit functions, as shown in Figure S9 and Figure S10. The extrapolation suggests a further reduction of X_{CH4} by 4.2 % to 7.1 % (absolute) due to the increase of the volume flow from 200 ml_N/min to 350 ml_N/min. This is in good agreement with a recent analysis conducted by Neuschitzer et al. [56]. They also observed exponential trends and describe a decrease of X_{CH4} by 3-4 % for every 0.1 slm the volume flow is increased up to $\dot{V}_{CH4} = 0.5$ slm.
The extrapolated (e.p.) methane conversions and hydrogen yields, which account for the volume flow and methane concentration effects, are depicted in Figure 4 of the main article for comparison. The positive uncertainties of these values refer to the difference between the estimated values (350 ml_N/min, 85 vol.% CH_4) and the 50 vol.% CH_4 values obtained with 200 ml_N/min on the third day of the respective pyrolysis conditions. Thus, the maximum deviation is obtained.
The potential influence of the carbon layer is depicted in the main article as the negative uncertainty of the estimated values to indicate that the absence of carbon powder on top of the liquid tin might have resulted in lower conversions and yields. The negative uncertainty refers to the difference between X_{CH4} or Y_{H2} on the third day of the respective pyrolysis condition and the extrapolated value for the first day of pyrolysis experiments. The differences obtained for a total PFG volume flow of 200 ml_N/min with 50 vol.% CH_4 and 100 vol.% CH_4 were compared, and the larger values were chosen as the negative uncertainties of the estimated conversions and yields.

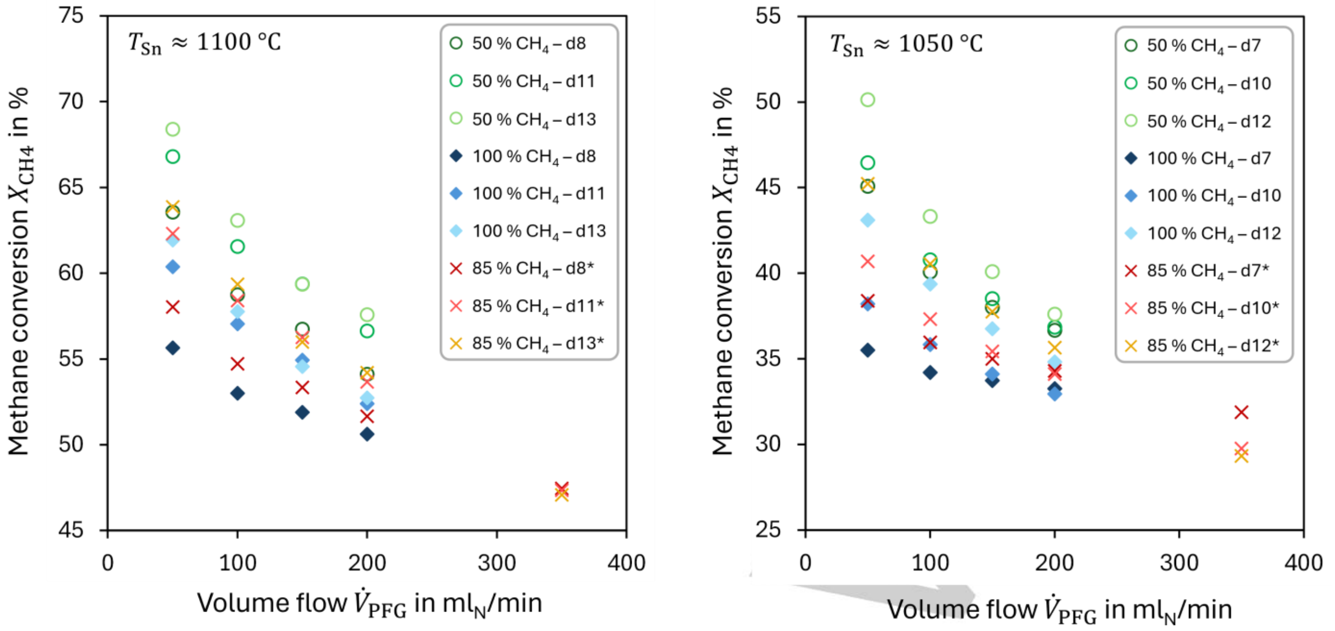

Figure S9. Effects of the volume flow of the pyrolysis feed gas and the initial methane concentration on methane conversion at 1100 °C and 1050 °C. The depicted data was obtained during previous experiments [47] with pure CH_4 and a 1:1 mixture of CH_4 and N_2 on several days (d7 to d13). The data was reevaluated for the present study. * indicates methane conversions interpolated for 85 vol. % CH_4 and extrapolated for a volume flow of 350 ml$_N$/min.

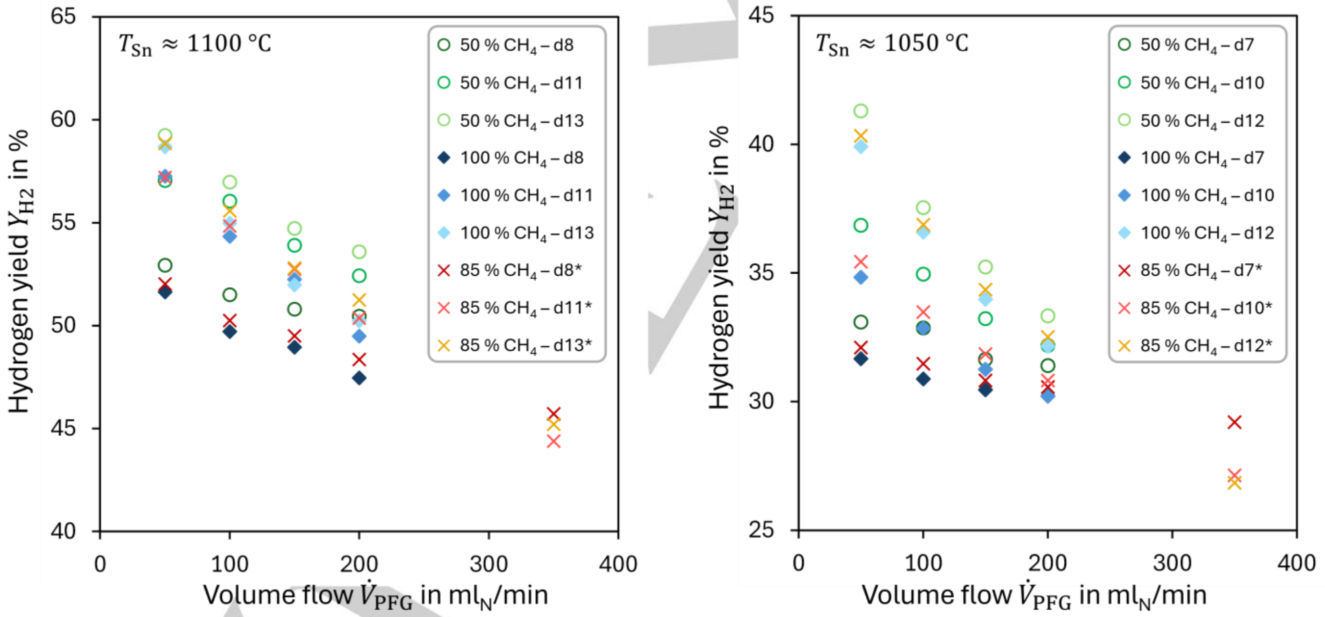

Figure S10. Effects of the volume flow of the pyrolysis feed gas and the initial methane concentration on hydrogen yield at 1100 °C and 1050 °C. The depicted data was obtained during previous experiments [47] with pure CH_4 and a 1:1 mixture of CH_4 and N_2 on several days (d7 to d13). The data was reevaluated for the present study. * indicates hydrogen yields interpolated for 85 vol. % CH_4 and extrapolated for a volume flow of 350 ml$_N$/min.

Analysis of Measurement Uncertainties

1 GC measurements of pyrolysis product gas and GST2 gas mixtures

1.1 Uncertainty of a single GC measurement

To check the calibration of the GC, a certified standard gas mixture (nominal volume fractions of 1 vol.% of H_2, N_2 and CH_4 in argon, actual values $y_{cert,i}$ given in certificate) with a relative uncertainty $u_{cert,rel}$ of 2 % was analyzed five times every morning before the start of pyrolysis experiments. These GC measurements of the volume fractions $y_{GC,i}$ were averaged and the standard deviation $u_{StdD,i}$ together with the difference $\Delta y_{\mathrm{cert,i}}$ of the averaged value to the certified value were used to determine the combined relative uncertainties $u_{C,rel}$ of a single GC measurement for H_2, N_2, CH_4 and Ar for the respective day as follows:

$$u_{\mathrm{C,rel}}(y_{i,\mathrm{GC}}) = \frac{\Delta y_{\mathrm{cert,i}} + u_{\mathrm{StdD},i} + u_{\mathrm{cert,rel}}(y_{i,\mathrm{cert}}) \cdot y_{\mathrm{cert,i}}}{y_{\mathrm{cert,i}}} \tag{S1}$$

As the calibration for C_2H_x (x = 2, 4, 6) components and CO_z (z = 1, 2) was not checked every morning, based on the calibration and previous experience with the detection of components close to the quantification limit a relative uncertainty of 30 % was assumed. For high concentrations of CH_4 and (relative to the calibrated range) high concentrations of CO_2, which were detected during the analysis of the gas mixture stored in GST2, a relative uncertainty of 10 % was assumed since high concentrations were not checked daily but calibrated high concentrations tend to have a lower relative uncertainty than very low concentrations. GC analysis of the gas mixture in GST2 revealed the presence of low concentrations of Ar. As Ar was only calibrated between 25 and 100 vol.%, a relative uncertainty of 100 % was assumed for low volume fractions (<10 vol.%). The following Table S8 lists the relative uncertainties of individual GC measurements for all gas volume fractions $y_{i,GC}$ analyzed during experiments #1-#4. No GC analysis was possible during experiment #4 as the pressure loss related to the low total volume flow was insufficient.

Table S8. Relative uncertainties of GC measurements of the listed gas volume fractions.

		Relative uncertainties $u_{C,rel}$ in %		
	Range in vol. %	#1	#2, #3	#4
$y_{H2,GC}$	≤ 5	3.61	2.60	3.70
$y_{N2,GC}$	≤ 5	8.12	7.93	10.54
$y_{CH4,GC}$	≤ 5	4.52	6.66	6.24
$y_{CH4,GC}$	80 - 100	10	10	10
$y_{Ar,GC}$	≤ 5	100	100	100
$y_{Ar,GC}$	90 - 100	1.89	0.23	1.48
$y_{C2Hx,GC}$	≤ 1	30	30	30
$y_{CO,GC}$	≤ 1	-	30	30
$y_{CO2,GC}$	≤ 1	-	30	30
$y_{CO2,GC}$	1 - 10	-	10	10

1.2 GC analysis of the pyrolysis product gas (PPG)

The volume fractions $y_{i,\mathrm{GC}}$ of CH_4, H_2, CO_2, CO, N_2, Ar and C_2H_x (with x = 2, 4, 6) in the pyroysis product gas, which was diluted with argon carrier gas, were determined via GC analysis. As no C_2H_6 was detected (see magnified chromatogram section in Figure S11), ethane is not considered in the following analysis.
As the sum of all eight volume fractions slightly exceeded 100 %, the measured volume fractions were corrected to yield 100 % in total:

$$y_{i,\mathrm{GCcorr}} = \frac{y_{i,\mathrm{GC}}}{\sum_{k=1}^{8} y_{k,\mathrm{GC}}} \tag{S2}$$

Uncertainty propagation analysis yields Equation S3 for the combined uncertainty of the corrected volume fractions $y_{i,\mathrm{GCcorr}}$. The combined uncertainties $u_{\mathrm{C}}(y_{i,\mathrm{GC}})$ of the measured volume fractions were obtained by multiplying the volume fraction $y_{i,\mathrm{GC}}$ with the respective relative uncertainty given in Table S8.

$$u_{\mathrm{C}}(y_{i,\mathrm{GCcorr}}) = \sqrt{\left(\frac{-y_{i,\mathrm{GC}}}{\left(\sum_{k=1}^{8} y_{k,\mathrm{GC}}\right)^2}\right)^2 \sum_{k=1,k\neq i}^{k=8} u_C^2(y_{k,\mathrm{GC}}) + \left(\frac{1}{\sum_{k=1}^{8} y_{k,\mathrm{GC}}} - \frac{y_{i,\mathrm{GC}}}{\left(\sum_{k=1}^{8} y_{k,\mathrm{GC}}\right)^2}\right)^2 u_C^2(y_{i,\mathrm{GC}})} \qquad \text{(S3)}$$

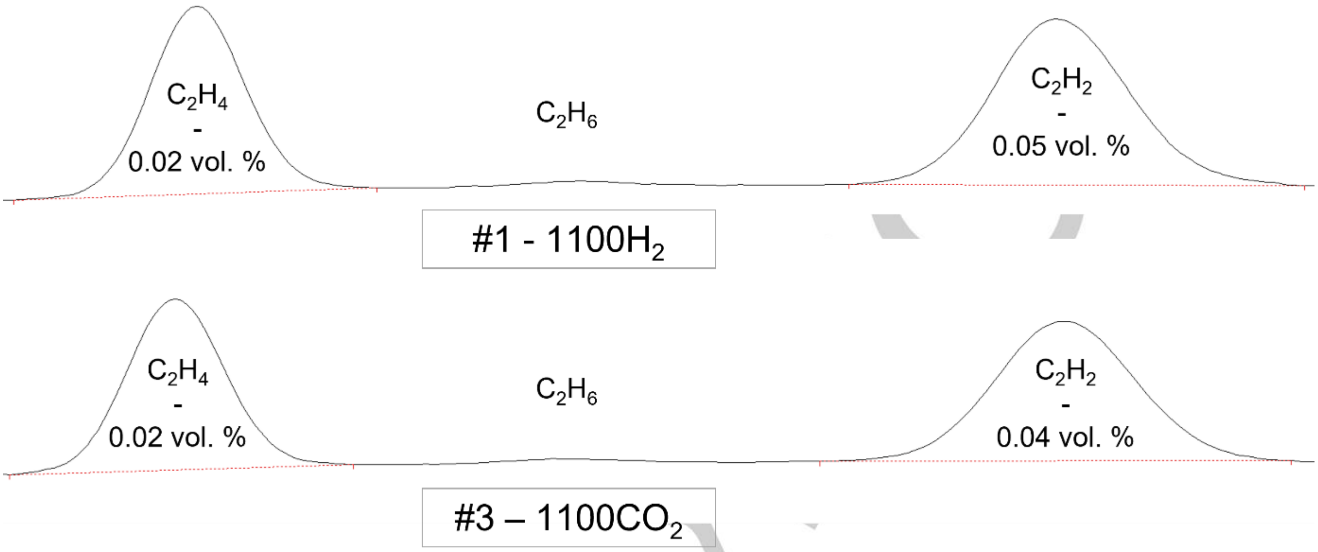

Figure S11. Strongly magnified section of representative chromatograms showing the lack of detectable amounts of C_2H_6. The limit of quantification for all permanent gases analyzed was given as 0.01 vol.% by the manufacturer.

For the quantification of the volume fractions $y_{i,\mathrm{PPG}}$ and volume flows $\dot{V}_{i,\mathrm{PPG}}$ of the individual components *i* of the pyrolysis product gas, in a first step, the known argon carrier gas flow $\dot{V}_{\mathrm{Ar}}$ was taken as a reference to calculate the total pyrolysis product gas (PPG) flow $\dot{V}_{\mathrm{PPG}}$ from the corrected argon volume fraction $y_{\mathrm{Ar,GCcorr}}$ via the following correlation:

$$y_{\mathrm{Ar,GCcorr}} = \frac{\dot{V}_{\mathrm{Ar}}}{\dot{V}_{\mathrm{Ar}} + \dot{V}_{\mathrm{PPG}}} \qquad \text{(S4)}$$

This yields Equation S5 for the PPG flow $\dot{V}_{\mathrm{PPG}}$:

$$\dot{V}_{\mathrm{PPG}} = \left(\frac{1}{y_{\mathrm{Ar,GCcorr}}} - 1\right)\dot{V}_{\mathrm{Ar}} \qquad \text{(S5)}$$

The volume fraction $y_{i,\mathrm{PPG}}$ of component *i* in the PPG (before dilution with argon) is given by Equation S6:

$$y_{i,\mathrm{PPG}} = \frac{\dot{V}_{\mathrm{i}}}{\dot{V}_{\mathrm{PPG}}} \qquad \text{(S6)}$$

Replacing $\dot{V}_{\mathrm{PPG}}$ in Equation S6 with Equation S5 yields Equation S7 for the volume fraction $y_{i,\mathrm{PPG}}$ of component *i* in the PPG before dilution, which is only based on (corrected) volume fractions measured via GC analysis:

$$y_{i,\mathrm{PPG}} = \frac{y_{i,\mathrm{GCcorr}}}{1 - y_{\mathrm{Ar,GCcorr}}} \qquad \text{(S7)}$$

The corresponding volume flow of component *i* is then given by Equation S8:

$$\dot{V}_{i,\mathrm{PPG}} = \frac{y_{i,\mathrm{GCcorr}}}{y_{\mathrm{Ar,GCcorr}}}\dot{V}_{\mathrm{Ar}} \qquad \text{(S8)}$$

Uncertainty propagation based on partial differentiation of Equation S7 and Equation S8 yields the combined uncertainty $u_C(y_{i,\mathrm{PPG}})$ of the volume fraction of component *i* in the PPG before dilution and the combined uncertainty $u_C(\dot{V}_{i,\mathrm{PPG}})$ of the respective volume flow:

$$u_C(y_{i,\mathrm{PPG}}) = \sqrt{\left(\frac{1}{1-y_{\mathrm{Ar,GCcorr}}}\right)^2 u_C^2(y_{i,\mathrm{GCcorr}}) + \left(\frac{y_{i,\mathrm{GCcorr}}}{\left(1-y_{\mathrm{Ar,GCcorr}}\right)^2}\right)^2 u_C^2(y_{\mathrm{Ar,GCcorr}})} \quad \text{(S9)}$$

$$u_C(\dot{V}_{i,\mathrm{PPG}}) = \sqrt{\left(\frac{y_{i,\mathrm{GCcorr}}}{y_{\mathrm{Ar,GCcorr}}}\right)^2 u_C^2(\dot{V}_{\mathrm{Ar}}) + \left(\frac{\dot{V}_{\mathrm{Ar}}}{y_{\mathrm{Ar,GCcorr}}}\right)^2 u_C^2(y_{i,\mathrm{GCcorr}}) + \left(\frac{-y_{i,\mathrm{GCcorr}}}{\left(y_{\mathrm{Ar,GCcorr}}\right)^2}\dot{V}_{\mathrm{Ar}}\right)^2 u_C^2(y_{\mathrm{Ar,GCcorr}})} \quad \text{(S10)}$$

The combined uncertainties $u_\mathrm{C}(y_{i,\mathrm{GCcorr}})$ of the gas volume fractions were calculated according to Equation S3. The argon volume flow $\dot{V}_{\mathrm{Ar}}$ was taken as the average of all values recorded from the process control system during a pyrolysis experiment at target process conditions. The argon mass flow controller had a relative uncertainty of 0.5 % for its entire control range. The uncertainty $u_C(\dot{V}_{\mathrm{Ar}})$ of the argon flow was calculated as the sum of the standard deviation determined from the recorded values and the absolute uncertainty obtained by multiplying the average value with the relative uncertainty.

As $n \geq 4$ GC measurements were carried out over the course of one pyrolysis experiment, the volume flows $\dot{V}_{i,\mathrm{PPG},j}$ of component *i* (with $1 \leq j \leq n$) were averaged in a next step to obtain the averaged volume flow $\dot{V}_{i,\mathrm{PPGav}}$ of component *i* during the pyrolysis experiment. The respective combined uncertainty $u_C(\dot{V}_{i,\mathrm{PPGav}})$ were calculated as the sum of the standard deviation $u_{std}(\dot{V}_{i,\mathrm{PPGav}})$ of the n volume flows and the maximum of their individual combined uncertainties as given by Equation S11:

$$u_\mathrm{C}(\dot{V}_{i,\mathrm{PPGav}}) = u_{std}(\dot{V}_{i,\mathrm{PPGav}}) + \max(u_C(\dot{V}_{i,\mathrm{PPG}})) \quad \text{(S11)}$$

1.3 GC analysis of the gas mixture stored in GST2 before and after pyrolysis experiments

During methane pyrolysis experiments the GC was needed to analyze the pyrolysis product gas. The gas mixture stored in the gas storage tank (GST2) was therefore only analyzed (*n* = 2 or 3 times) by GC before and after pyrolysis experiments. In a first step the measured volume fractions $y_{i,\mathrm{GST2}}$ were averaged for the *n* GC measurements to obtain the averaged volume fractions $\bar{y}_{i,\mathrm{GST2}}$.

The combined uncertainties $u_\mathrm{C}(\bar{y}_{i,\mathrm{GST2}})$ of the thus determined averaged volume fractions $\bar{y}_{i,\mathrm{GST2}}$ were calculated via partial differentiation to consider uncertainty propagation as follows:

$$u_\mathrm{C}(\bar{y}_{i,\mathrm{GST2}}) = \sqrt{\left(\frac{1}{n}\right)^2 \sum_{j=1}^{j=n} \left(u_\mathrm{C}(y_{i,\mathrm{GST2},j})\right)^2} \quad \text{(S12)}$$

The combined uncertainties $u_\mathrm{C}(y_{i,\mathrm{GST2},j})$ of the measured volume fractions were obtained by multiplying the volume fractions $y_{i,\mathrm{GST2},j}$ of the *j*-th GC measurement with the respective relative uncertainty given in Table S8.

As the sum of all six averaged volume fractions $\bar{y}_{i,\mathrm{GST2}}$ slightly exceeded 100 %, the volume fractions were corrected to yield 100 % in total:

$$\bar{y}_{i,\mathrm{GST2corr}} = \frac{\bar{y}_{i,\mathrm{GST2}}}{\sum_{k=1}^{6} \bar{y}_{k,\mathrm{GST2}}} \quad \text{(S13)}$$

Uncertainty propagation analysis yields Equation S14 for the combined uncertainty of the corrected averaged volume fractions $\bar{y}_{i,\mathrm{GST2corr}}$.

$$u_\mathrm{C}(\bar{y}_{i,\mathrm{GST2corr}}) = \sqrt{\left(\frac{-\bar{y}_{i,\mathrm{GST2}}}{\left(\sum_{k=1}^{6} \bar{y}_{k,\mathrm{GST2}}\right)^2}\right)^2 \sum_{k=1,k\neq i}^{k=6} u_C^2(\bar{y}_{k,\mathrm{GST2}}) + \left(\frac{1}{\sum_{k=1}^{6} \bar{y}_{k,\mathrm{GST2}}} - \frac{\bar{y}_{i,\mathrm{GST2}}}{\left(\sum_{k=1}^{6} \bar{y}_{k,\mathrm{GST2}}\right)^2}\right) u_C^2(\bar{y}_{i,\mathrm{GST2}})} \quad \text{(S14)}$$

WILEY-VCH

2 Methanation Product Gas

2.1 Gas volume fractions of the methanation product gas (MPG)

H_2, CO_2, CO and CH_4 were measured with an IR analyzer, as described in the experimental section of the main article. The analyzer uncertainties are given below in Table S9. For experiment #1 with hydrogen surplus, the measured hydrogen volume fraction $y_{H2,MPG}$ was corrected for a known systematic deviation of -0.75 vol.%, which was also considered in the combined uncertainty of the hydrogen measurement. For all other experiments with CO_2 surplus, the methanation product gas analyzer did not detect any hydrogen. The gas chromatograph (GC) analysis of several samples taken from the gas storage tank (GST2) before and after pyrolysis experiments detected hydrogen, though. Therefore, the GC hydrogen volume fractions were included in the methanation product gas composition. GC analysis is more sensitive to low hydrogen concentrations and the corresponding analysis of GC measurement uncertainty (see sections 1.1 and 1.3) was included in the uncertainty analysis for the methanation product gas. Additionally, also the relative standard deviation of $\bar{y}_{\mathrm{CO_2,MPG}}$, the average CO_2 volume fraction determined via IR analysis, was included in the combined uncertainty $u_{\mathrm{C}}(\bar{y}_{\mathrm{H_2,GC-MPG}})$ of the average hydrogen volume fraction in the methanation product gas determined via GC analysis. This assumes that the volume fraction of H_2 follows similar relative fluctuations as its methanation reaction partner CO_2. However, GC analysis only determines the hydrogen volume fraction at the beginning and/or end of the experiment and can therefore not provide a standard deviation of the volume fraction for the entire period of interest. $u_{\mathrm{C}}(\bar{y}_{\mathrm{H_2,GC-MPG}})$ is therefore calculated as follows:

$$u_{\mathrm{C}}(\bar{y}_{\mathrm{H_2,GC-MPG}}) = u_{\mathrm{C}}(\bar{y}_{\mathrm{H_2,GST2corr}}) + \bar{y}_{\mathrm{H_2,GST2corr}} \frac{u_{\mathrm{C}}(\bar{y}_{\mathrm{CO_2,MPG}})}{\bar{y}_{\mathrm{CO_2,MPG}}} \tag{S15}$$

A mixture of unknown gases (nitrogen and potentially argon, maybe also others) was assumed to make up the undetected remaining volume fraction $y_{\mathrm{div,MPG}}$, which was determined by difference. The standard deviation $u_{\mathrm{StdD},i}$ of all measurements taken within the relevant time periods for components *i* was added to the combined uncertainty of the respective components.

Table S9. Measurement uncertainties of the methanation product gas (MPG) characterization

Uncertainties	$y_{\mathrm{H2,MPG}}$ / vol.%	$y_{\mathrm{CO2,MPG}}$ / vol.%	$y_{\mathrm{CO,MPG}}$ / vol.%	$y_{\mathrm{CH4,MPG}}$ / vol.%	$y_{\mathrm{div,MPG}}$ / vol.%
Systemic u_{sys}	-0.75				
Analyser u_{A}	0.25	0.075	0.075	0.075	
Combined u_{C}	1.0 + $u_{\mathrm{StD,H2}}$ or $u_{\mathrm{C}}(\bar{y}_{\mathrm{H_2,GC-MPG}})$	0.075 + $u_{\mathrm{StD,CO2}}$	0.075 + $u_{\mathrm{StD,CO}}$	0.075 + $u_{\mathrm{StD,CH4}}$	$\sum u_{\mathrm{C}}(y_k)$ for k = CH_4, H_2, CO_2, CO

2.2 Reactant gas volume flow from GST2 to the pyrolysis reactor

Based on the average composition of the MPG during the pyrolysis time period, the volume flow from GST2 to the pyrolysis reactor (pyrolysis feed gas, PFG) was corrected using the manufacturer's online tool FLUIDAT.[57] As the mass flow controller (MFC) was calibrated for pure methane, a mixture of methane and other gases resulted in slightly different volume flows. The volume flows recorded from the control system (set value 350 $\mathrm{ml_N\ min^{-1}}$ with index N referring to 1.01325 bar and 273.15 K in the context of gas volume) were therefore corrected by the calculated percentage given in Table S10 as $u_{\dot{V}_{\mathrm{PFG,corr,rel}}}$ to obtain $\dot{V}_{\mathrm{PFG,corr}}(t)$ at time t and then averaged over the relevant time period. The combined uncertainty of the averaged values was calculated as the sum of the standard deviation of the corrected values and the uncertainty obtained by multiplying the average value with the sum of the relative uncertainty of the MFC $u_{\mathrm{MFC,rel}}$ and the flow correction $u_{\dot{V}_{\mathrm{PFG,corr,rel}}}$, as shown in Table S10. For experiment #1, which lasted about four hours and where the MPG composition varied a lot more than in the other experiments, the flow correction was calculated both for the maximum (14.7 vol.%) and the minimum (2.5 vol.%) content of hydrogen during the respective time period. $\dot{V}_{\mathrm{PFGav,corr}}$ was obtained with a +4 % correction in experiment #1, which

was the average of the minimum and the maximum correction. However, the uncertainty $u_{\dot{V}_{\mathrm{PFG,corr,rel}}}$ given in Table S10 for experiment #1 is based on the 'worst case' maximum correction of 6.5 % corresponding to the highest observed hydrogen content.

Table S10. Uncertainties of the corrected volume flows that account for gas mixtures instead of pure methane.

	#1	#2	#3	#4
$\dot{V}_{\mathrm{PFGav,corr}}$ /ml$_N$ min^{-1}	364.0	354.9	358.0	353.5
Std. dev. /ml$_N$ min^{-1}	0.1	0.1	0.1	0.1
$u_{\mathrm{MFC,rel}}$ / %	0.5	0.5	0.5	0.5
$u_{\dot{V}\mathrm{PFG,corr,rel}}$ / %	6.5	1.4	2.3	1.0
Combined u_C /ml$_N$ min^{-1}	25.6	6.9	10.1	5.4

Next, the volume of GST2 was divided by the corrected volume flows given in Table S10 to obtain an approximation of the time lag between the methanation and the pyrolysis unit. The underlying assumption of plug flow behavior of the gas inside GST2 is based on a comparison of the temporal fluctuation of the $y_{\mathrm{div,MPG}}$ and the $y_{\mathrm{N2,PPG}}$ volume fractions shown below in Figure S12 for experiment #1, where the observed and the calculated time lag match well. Differences in the absolute values of the volume fractions shown in Figure S12 are probably due to several reasons: $y_{\mathrm{div,MPG}}$ represents the sum of undetected gas species in the methanation product gas. This encloses N_2 but possibly also H_2O and Ar.

The reactions expected to dominate within the pyrolysis reactor result in an overall increase of gas molecules (see theoretical background in the main article), which would reduce the measured nitrogen volume fraction in the pyrolysis product gas compared to the methanation product gas.

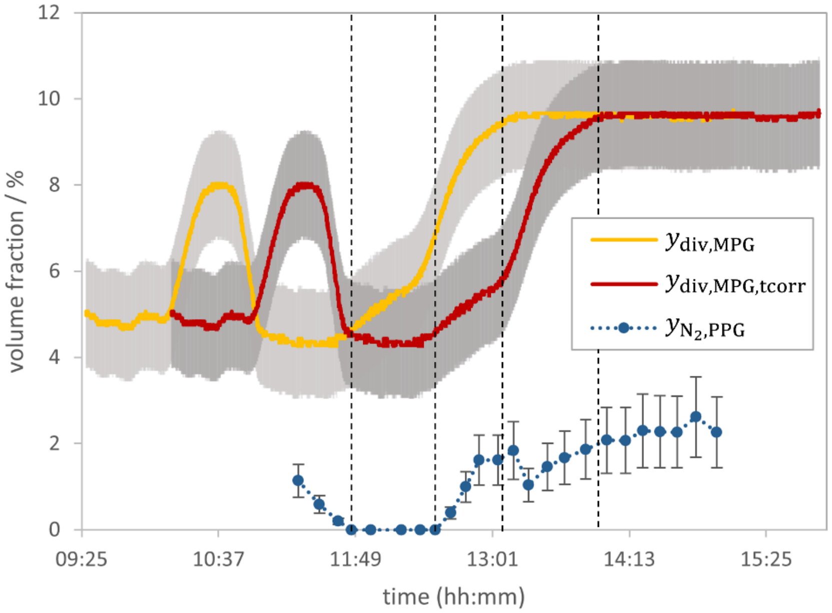

Figure S12. Time lag caused by the gas storage tank (GST2) between the methanation unit and the pyrolysis unit. Time correction based on plug flow assumption for GST2.

The average volume fractions of the pyrolysis feed gas $\bar{y}_{i,\mathrm{MPG}}$ reported in Table S8 were therefore obtained from the MPG analysis after accounting for the calculated time lag. The respective combined uncertainties were calculated as described in section 2.1. The corrected gas volume flow $\dot{V}_{\mathrm{PFGav,corr}}$ was not further corrected iteratively as the thus obtained averaged volume fractions did hardly deviate from the compositions considered for the volume flow corrections of experiments #2-#4. In the case of experiment #1, the thus obtained averaged volume fractions were within the range considered (see above). Therefore, also for experiment #1 we decided against an iterative volume flow correction.

The average volume flow $\dot{V}_{i,\mathrm{PFGav}}$ of each component *i* into the pyrolysis reactor was calculated by multiplying the total corrected and averaged volume flow $\dot{V}_{\mathrm{PFGav,corr}}$ with the averaged volume fraction $\bar{y}_{i,\mathrm{MPG}}$ of component *i*. The combined uncertainty $u_{\mathrm{C}}(\dot{V}_{i,\mathrm{PFGav}})$ is then given by:

$$u_{\mathrm{C}}(\dot{V}_{i,\mathrm{PFGav}}) = \sqrt{\left(\dot{V}_{\mathrm{PFGav,corr}}\right)^2 u_C^2(\bar{y}_{i,\mathrm{MPG}}) + \left(\bar{y}_{i,\mathrm{MPG}}\right)^2 u_C^2(\dot{V}_{\mathrm{PFGav,corr}})} \tag{S16}$$

3 Characterization of the Process Performance

3.1 Conversion, yield and selectivity of gas phase components

Assuming ideal gas behavior of the gas phase components, the conversions, yields and selectivities were calculated based on volume flows of components a (product) and i (reactant). Equation S17 describes the conversion X_i (with i = CH_4 or CO_2):

$$X_i = \frac{\dot{V}_{i,\mathrm{PFGav}} - \dot{V}_{i,\mathrm{PPGav}}}{\dot{V}_{i,\mathrm{PFGav}}} \tag{S17}$$

The combined uncertainty of the conversion is then given by Equation S18 as follows:

$$u_{\mathrm{C}}(X_i) = \sqrt{\left(\frac{\dot{V}_{i,\mathrm{PPGav}}}{\dot{V}_{i,\mathrm{PFGav}}^2}\right)^2 u_C^2(\dot{V}_{i,\mathrm{PFGav}}) + \left(\frac{1}{\dot{V}_{i,\mathrm{PFGav}}}\right)^2 u_C^2(\dot{V}_{i,\mathrm{PPGav}})} \tag{S18}$$

The yield $Y_{a,i}$ of product a (with a = H_2 or CO, when i = CH_4 or CO_2 respectively) was calculated according to Equation S19. The underlying stoichiometries correspond to the methane pyrolysis reaction (Equation 3, main article) and the CO_2 carbon gasification reaction (Equation 4, main article).

$$Y_{a,i} = 0.5\frac{\dot{V}_{a,\mathrm{PPGav}} - \dot{V}_{a,\mathrm{PFGav}}}{\dot{V}_{i,\mathrm{PFGav}}} \tag{S19}$$

The combined uncertainty of the yield $u_{\mathrm{C}}(Y_{a,i})$ is obtained according to Equation S20:

$$u_{\mathrm{C}}(Y_{a,i}) = \sqrt{\left(\frac{0.5}{\dot{V}_{i,\mathrm{PFGav}}}\right)^2 u_C^2(\dot{V}_{a,\mathrm{PPGav}}) + \left(\frac{-0.5}{\dot{V}_{i,\mathrm{PFGav}}}\right)^2 u_C^2(\dot{V}_{a,\mathrm{PFGav}}) + \left(-0.5\frac{\dot{V}_{a,\mathrm{PPGav}} - \dot{V}_{a,\mathrm{PFGav}}}{(\dot{V}_{i,\mathrm{PFGav}})^2}\right)^2 u_C^2(\dot{V}_{i,\mathrm{PFGav}})} \tag{S20}$$

Equation S21 describes the selectivity $S_{a,i}$ of a product a (with a = H_2 or CO, when i = CH_4 or CO_2 respectively):

$$S_{a,i} = 0.5\frac{\dot{V}_{a,\mathrm{PPGav}} - \dot{V}_{a,\mathrm{PFGav}}}{\dot{V}_{i,\mathrm{PFGav}} - \dot{V}_{i,\mathrm{PPGav}}} \tag{S21}$$

The combined uncertainty of the selectivity $u_{\mathrm{C}}(S_{a,i})$ is then given by Equation S22:

$$u_{\mathrm{C}}(S_{a,i}) = \sqrt{\left(\left|\frac{0.5}{\dot{V}_{i,\mathrm{PFGav}} - \dot{V}_{i,\mathrm{PPGav}}}\right|\right)^2 \left(u_C^2(\dot{V}_{a,\mathrm{PPGav}}) + u_C^2(\dot{V}_{a,\mathrm{PFGav}})\right) + \left(\left|\frac{0.5(\dot{V}_{a,\mathrm{PPGav}} - \dot{V}_{a,\mathrm{PFGav}})}{\left(\dot{V}_{i,\mathrm{PFGav}} - \dot{V}_{i,\mathrm{PPGav}}\right)^2}\right|\right)^2 \left(u_C^2(\dot{V}_{i,\mathrm{PPGav}}) + u_C^2(\dot{V}_{i,\mathrm{PFGav}})\right)} \tag{S22}$$

3.2 Integral solid carbon yield

3.2.1 Integral carbon feed of the pyrolysis reactor

The integral methane volume fed into the pyrolysis reactor was calculated to derive the integral mass of carbon m_{C} available for carbon formation during each of the pyrolysis experiments. Carbon atoms contained in the feed gas as CO or CO_2 were not considered as sources for solid carbon formation. CO volume fractions of the MPG were very low in all experiments and furthermore, the equilibrium of the Boudouard reaction is on the side of CO for the pyrolysis temperatures considered (see Figure 7 c in the main article). CO_2 might therefore consume solid carbon,

WILEY-VCH

counteracting its formation from methane. An integral approach was required as the total mass of the solid carbon product was determined by weighing at the end of each pyrolysis experiment.
In a first step, the total pyrolysis feed gas (PFG) volume $V_{\mathrm{PFG,int}}$ for each experiment was calculated by integrating the corrected (according to section 2.2.) time resolved gas volume flows $\dot{V}_{\mathrm{PFG,corr}}(t)$ over the respective pyrolysis period according to Equation S23. $V_{\mathrm{PFG,int}}$ also includes the ramp up and ramp down time periods at the start and the end of a pyrolysis experiment when nitrogen was slowly replaced by the reactant gas and vice versa. This differs from the gas phase analysis (methane conversion, hydrogen yield, etc.), for which only the time periods at target gas composition were considered. GC analysis of the pyrolysis product gas was only done under those target conditions. However, carbon formation also takes place during the switching from nitrogen to reactant gas and back. Since the carbon yield can only be determined as an integral value with our experimental setup, the gas flow into the reactor during transient states has to be considered as well to calculate the carbon yield.

$$V_{\mathrm{PFG,int}} = \sum_{t=0}^{t=n} (\dot{V}_{\mathrm{PFG,corr}}(t)\Delta t) \qquad \text{(S23)}$$

Over the entire period of the pyrolysis experiment a dataset with a time resolution of $\Delta t = 1\ \mathrm{s}$ was recorded, resulting in a total of n values for each recorded variable. Considering uncertainty propagation, the combined uncertainty $u_C(V_{\mathrm{PFG,int}})$ was calculated according to Equation S24:

$$u_C(V_{\mathrm{PFG,int}}) = \sqrt{\sum_{t=0}^{t=n} \Delta t^2\, u_C^2(\dot{V}_{\mathrm{PFG,corr}}(t))} \qquad \text{(S24)}$$

The uncertainty $u_C(\dot{V}_{\mathrm{PFG,corr}}(t))$ was obtained by multiplying $\dot{V}_{\mathrm{PFG,corr}}(t)$ with the sum of the relative uncertainty of the MFC $u_{\mathrm{MFC,rel}}$ and the relative uncertainty of the volume flow correction $u_{\dot{V}_{\mathrm{PFG,corr,rel}}}$, which are both given in Table S10 for each of the experiments.
The total volume of methane $V_{\mathrm{CH4,PFG}}$ fed into the pyrolysis reactor during a pyrolysis experiment was calculated by multiplying the total pyrolysis feed gas volume $V_{\mathrm{PFG,int}}$ with the corrected, averaged methane volume fraction of the methanation product gas $\bar{y}_{\mathrm{MPG,CH4}}$ (section 2). The total mass of available carbon $m_{\mathrm{C,PFG}}$ introduced into the pyrolysis reactor as CH_4 was then obtained under the assumption of ideal gas behavior by dividing $V_{\mathrm{CH4,PFG}}$ by the molar gas volume v_{m} (at 1.01325 bar and 273.15 K) and multiplying with the molar weight of carbon $\widetilde{\mathrm{M}}_{\mathrm{C}}$ (12.0106 g mol^{-1} with an uncertainty $u(\widetilde{\mathrm{M}}_{\mathrm{C}})$=0.001 g mol^{-1} [58]):

$$m_{\mathrm{C,PFG}} = \frac{V_{\mathrm{PFG,int}} \cdot \bar{y}_{\mathrm{MPG,CH4}} \cdot \widetilde{\mathrm{M}}_{\mathrm{C}}}{v_{\mathrm{m}}} \qquad \text{(S25)}$$

Neglecting the uncertainty of the molar gas volume as ideal gas behavior was assumed, Equation S26 describes the combined uncertainty of the available carbon mass fed into the pyrolysis reactor during one experiment:

$$u_C(m_{\mathrm{C,PFG}}) = \sqrt{\left(\frac{\bar{y}_{\mathrm{MPG,CH4}} \cdot \widetilde{\mathrm{M}}_{\mathrm{C}}}{v_{\mathrm{m}}}\right)^2 u_C^2(V_{\mathrm{PFG,int}}) + \left(\frac{V_{\mathrm{PFG,int}} \cdot \widetilde{\mathrm{M}}_{\mathrm{C}}}{v_{\mathrm{m}}}\right)^2 u_C^2(\bar{y}_{\mathrm{MPG,CH4}}) + \left(\frac{V_{\mathrm{PFG,int}} \cdot \bar{y}_{\mathrm{MPG,CH4}}}{v_{\mathrm{m}}}\right)^2 u_C^2(\widetilde{\mathrm{M}}_{\mathrm{C}})} \qquad \text{(S26)}$$

3.2.2 Integral mass of solid carbon products

The solid products obtained during a pyrolysis experiment were carried out of the pyrolysis reactor and collected downstream in a particle filter. Once a synthesis experiment ended, the filter bag(s) containing the solid products (carbon and tin) were weighed to determine the mass difference to the empty filter bag(s), which were weighed before the start of the experiment. Additional glass bottles were used in some experiments to collect carbon powder sticking to the filter inlet and the product mass was also determined by difference. The scales used had an uncertainty of 0.01 g and were calibrated with a certified balancing weight (200 g) every morning.
As the masses were only measured once, the uncertainty of the scales was multiplied by three to account for the uncertainty of a single mass measurement. As the mass of the total solid pyrolysis product $m_{\mathrm{tot,PPs}}$ was obtained from several mass measurements (two for single filter bag – empty and full, >2 if multiple filter bags and/ or bottles were used), the uncertainty of 0.03 g was multiplied by the amount of mass measurements. For example, in experiment #2 where a single filter bag was used, the uncertainty of the product mass was determined as 0.06 g, as the empty and the full filter bag had to be weighed.

The carbon fraction of the collected solid product was determined via CHN elemental analysis. A sample was taken from the total collected product and then analyzed several times with a microanalyzer. The uncertainty $u(y_{\mathrm{C,PPs}})$ of the carbon mass fraction of the solid pyrolysis product $y_{\mathrm{C,PPs}}$ was calculated as the sum of the standard deviation and the detection limit of 0.2 wt.%.
Multiplying the total solid pyrolysis product $m_{\mathrm{tot,PPs}}$ with the carbon mass fraction $y_{\mathrm{C,PPs}}$ yields the mass of the carbon product $m_{\mathrm{C,PPs}}$ synthesized during a pyrolysis experiment. Equation S27 gives the combined uncertainty of $m_{\mathrm{C,PPs}}$.

$$u_C(m_{\mathrm{C,PPs}}) = \sqrt{\left(y_{\mathrm{C,PPs}}\right)^2 u_C^2\left(m_{\mathrm{tot,PPs}}\right) + \left(m_{\mathrm{tot,PPs}}\right)^2 u_C^2\left(y_{\mathrm{C,PPs}}\right)} \tag{S27}$$

3.2.3 Solid carbon yield

The integral carbon yield $Y_{\mathrm{C,CH_4}}$ of a pyrolysis experiment was obtained by dividing the mass of the solid carbon product $m_{\mathrm{C,PPs}}$ by the available mass of carbon $m_{\mathrm{C,PFG}}$ contained in the pyrolysis feed gas as CH_4. The uncertainty of the carbon yield was then calculated according to Equation S28.

$$u_C(Y_{\mathrm{C,CH_4}}) = \sqrt{\left(\frac{1}{m_{\mathrm{C,PFG}}}\right)^2 u_C^2\left(m_{\mathrm{C,PPs}}\right) + \left(\frac{-m_{\mathrm{C,PPs}}}{m_{\mathrm{C,PFG}}^2}\right)^2 u_C^2\left(m_{\mathrm{C,PFG}}\right)} \tag{S28}$$

The selectivity $S_{\mathrm{C,CH_4}}$ was not calculated by dividing the carbon yield $Y_{\mathrm{C,CH_4}}$ by the methane conversion $X_{\mathrm{CH_4}}$. As elaborated previously, the reference period for $X_{\mathrm{CH_4}}$ only includes the steady-state period when the target reactant gas flow was reached. The integral carbon yield $Y_{\mathrm{C,CH_4}}$ on the other hand also includes ramp-up and ramp-down time when a mixture of nitrogen and reactant gas was fed into the pyrolysis reactor.

References

[46] T. G. Geißler, *Doctoral Thesis*, Karlsruhe Institute of Technology, **2017**
[48] N. Uhlenbruck, B. Dietrich, C. Hofberger, L. Stoppel, T. Wetzel, *Energy Technol.,* **2022***, 10,* 2200654. DOI: 10.1002/ente.202200654
[56] D. Neuschitzer, D. Scheiblehner, H. Antrekowitsch, S. Wibner, A. Sprung, *Energies*, **2023**, *16*, 7058. DOI: 10.3390/en16207058
[57] Bronkhorst, 'FLUIDAT', https://www.fluidat.com/default.asp , accessed Nov **2023**
[58] M. E. Wieser, T. B. Coplen, *Pure Appl. Chem.,* **2010***, 83*, 359-396. DOI: 10.1351/pac-rep-10-09-14

WILEY-VCH
